# Microplastics dampen the self-renewal of hematopoietic stem cells by disrupting the gut microbiota-hypoxanthine-Wnt axis

**DOI:** 10.1038/s41421-024-00665-0

**Published:** 2024-03-29

**Authors:** Lingli Jiang, Yishan Ye, Yingli Han, Qiwei Wang, Huan Lu, Jinxin Li, Wenchang Qian, Xin Zeng, Zhaoru Zhang, Yanmin Zhao, Jimin Shi, Yi Luo, Yunfei Qiu, Jun Sun, Jinghao Sheng, He Huang, Pengxu Qian

**Affiliations:** 1 Center for Stem Cell and Regenerative Medicine and Bone Marrow Transplantation Center of the First Affiliated Hospital, Zhejiang University School of Medicine, Hangzhou, Zhejiang China; 2 Liangzhu Laboratory, Zhejiang University, Hangzhou, Zhejiang China; 3 Institute of Hematology, Zhejiang University & Zhejiang Engineering Laboratory for Stem Cell and Immunotherapy, Hangzhou, Zhejiang China; 4 Bone Marrow Transplantation Center, The First Affiliated Hospital, Zhejiang University School of Medicine, Hangzhou, Zhejiang China

**Keywords:** Haematopoietic stem cells, Metabolomics, Cell biology

## Abstract

Microplastics (MPs) are contaminants ubiquitously found in the global biosphere that enter the body through inhalation or ingestion, posing significant risks to human health. Recent studies emerge that MPs are present in the bone marrow and damage the hematopoietic system. However, it remains largely elusive about the specific mechanisms by which MPs affect hematopoietic stem cells (HSCs) and their clinical relevance in HSC transplantation (HSCT). Here, we established a long-term MPs intake mouse model and found that MPs caused severe damage to the hematopoietic system. Oral gavage administration of MPs or fecal transplantation of microbiota from MPs-treated mice markedly undermined the self-renewal and reconstitution capacities of HSCs. Mechanistically, MPs did not directly kill HSCs but disrupted gut structure and permeability, which eventually ameliorated the abundance of *Rikenellaceae* and hypoxanthine in the intestine and inactivated the HPRT-Wnt signaling in bone marrow HSCs. Furthermore, administration of *Rikenellaceae* or hypoxanthine in mice as well as treatment of WNT10A in the culture system substantially rescued the MPs-induced HSC defects. Finally, we validated in a cohort of human patients receiving allogenic HSCT from healthy donors, and revealed that the survival time of patients was negatively correlated with levels of MPs, while positively with the abundance of *Rikenellaceae*, and hypoxanthine in the HSC donors’ feces and blood. Overall, our study unleashes the detrimental roles and mechanisms of MPs in HSCs, which provides potential strategies to prevent hematopoietic damage from MPs and serves as a fundamental critique for selecting suitable donors for HSCT in clinical practice.

## Introduction

Hematopoietic stem cells (HSCs) supply the blood system with 280 billion cells every day^1^, which are attributed to their capacities of self-renewal and multilineage differentiation potential^2^. Due to their long-term reconstitution potential, HSC transplantation (HSCT) has become a curative therapy for various types of blood and immune disorders^3^. Thus, it is important to delineate the regulatory mechanisms underlying their self-renewal ability in a steady state and under stress conditions. Recent studies have shown that environmental factors, including sedentary lifestyle, tobacco use, alcohol intake, dietary metabolites, air pollutants, etc., play pivotal roles in deteriorating HSC self-renewal^4–8^ and are a risk factor for reconstitution failure and graft-versus-host disease (GvHD) after allogeneic HSCT^9^. However, it remains largely elusive about the roles and mechanisms of these environmental factors in the regulation of hematopoiesis.

Microplastics (MPs), defined as plastic fibers, particles, or films under 5 mm in length, arise from plastic waste fragmentation or are purposefully manufactured for special applications, which are new types of environmental contaminants found throughout the world’s ecosystems^10,11^. Humans inevitably intake MPs by diet, inhalation, or dermal contact, and a recent study estimates that we ingest 0.1–5 g of MPs a week on average^12^, which may have toxicity issues to human health^13,14^. Indeed, several studies have detected MPs in human stools^15^, lung tissues^16^, and the blood^17^. Microplastic exposure causes oxidative stress and inflammation in the digestive, respiratory and reproductive systems^13^, and also perturbs the intestinal microbiota and metabolites^18,19^. Recently, several studies reported that MPs disrupt the homeostasis of the gut microbiota, metabolism, and inflammation, which eventually induce hematopoietic damages^20,21^. Nonetheless, there is still a lack of a comprehensive understanding of the roles and mechanisms of MPs in HSC self-renewal and the clinical outcomes in patients who have received HSCT.

The intestinal microbiota has emerged as a vital modulator of metabolic, immune system^22,23^, inflammatory^24^, aging^25,26^ and neuropsychiatric disorders^27^. Furthermore, microbiota density, biodiversity and species composition of recipients are linked to treatment-related mortality, infections, and organ failure after HSCT^28^. Indeed, previous studies have revealed that gut microbiota and metabolites can protect the hematopoietic system from damage by ionizing radiation^29^, and also found a microbiota-macrophage-iron axis under stress conditions in the hematopoietic system^30^. However, the detailed mechanisms by which the microbiota affects hematopoiesis remain to be elucidated, and more evidence on the relationship between the environmental pollutants and gut microbiome in HSCs is needed.

## Results

### Long-term ingestion of MPs impairs the hematopoietic system

To assess the biological effect of MPs on the hematopoietic system, we initially chose commercially available MPs with wide application, such as polystyrene (PS), polymethyl methacrylate (PMMA) and polyethylene (PE). We revealed the spherical-shaped particles with an average size of about 500 nm (Supplementary Fig. S1a–d), and about 600–850 nm measured in double distilled water by dynamic light scattering (Supplementary Fig. S1e–i). The Zeta potential of the MPs showed a slightly anionic surface charge of approximately −5 to −14 mV (Supplementary Fig. S1j–n). To investigate the distribution of MPs in vivo, we used an indocyanine green labeled polystyrene (ICG-PS) mouse model (Supplementary Fig. S2a). The labeling of ICG did not affect the physical properties of PS MPs (Supplementary Fig. S1b, f, k), and they were predominantly enriched in the gastrointestinal tissues, slightly distributed in the kidney, but not found in the other organs (Supplementary Fig. S2b–e). Moreover, in the peripheral blood, we observed high fluorescence intensity in the high-dose group compared to the control or low-dose groups (Supplementary Fig. S2f), supporting the notion that MPs can enter the bloodstream from the intestine^17^. Observing MPs in the gastrointestinal tissues and peripheral blood of mice supported in-depth research into the relationship between MPs and gastrointestinal tissues as well as the hematopoietic system.

**Fig. 1:**
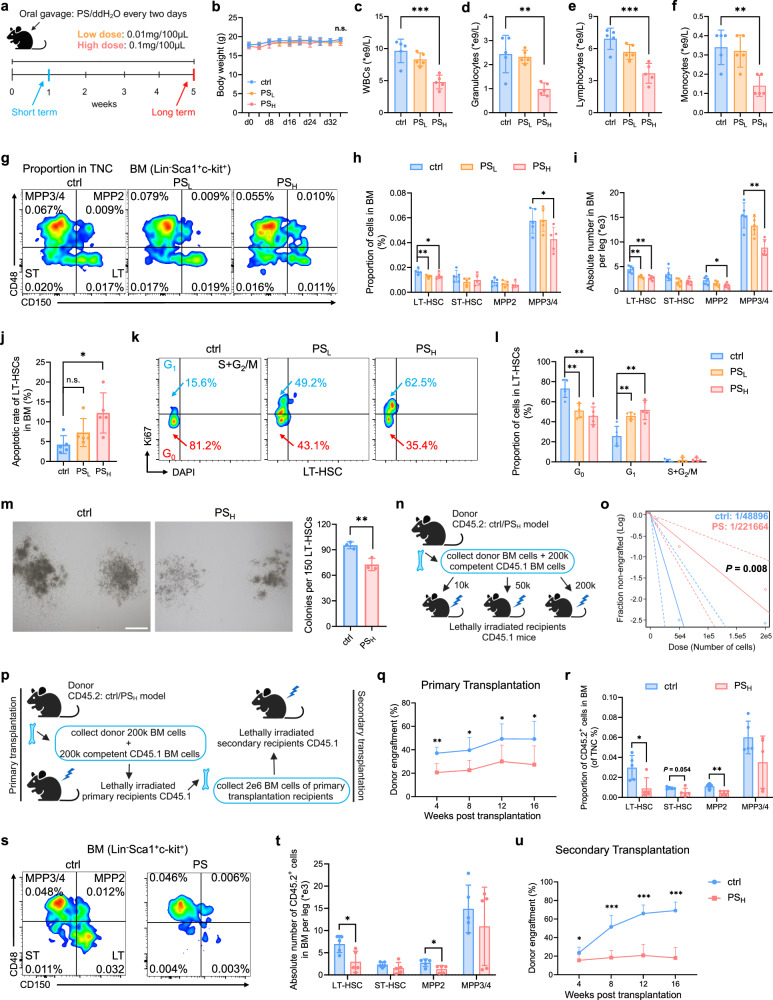
Long-term ingestion of microplastics results in severe damage to the hematopoietic system. **a** Schematic outlining mouse model for MPs-treatment (*n* = 5 per group). **b** Body weight of each mouse of the three groups. PS_L_, 0.01 mg/100 μL; PS_H_, 0.1 mg/100 μL. **c**–**f** Number of white blood cells (WBCs) (**c**), granulocytes (**d**), lymphocytes (**e**) and monocytes (**f**) in peripheral blood. **g** Representative flow cytometry images of the LSK compartment (Lin^–^Sca-1^+^c-Kit^+^) showing LT-HSC, ST-HSC, MPP2 and MPP3/4 population frequencies within total bone marrow (BM). **h**, **i** Proportion of cells in BM (**h**) and absolute cell number in BM (**i**). **j** Apoptosis in LT-HSCs. Representative flow cytometry images of cell cycle distribution (**k**) and proportion of cells synchronized at the G_0_, G_1_ and S + G_2_/M phases (**l**) within LT-HSCs. **m** Representative images of CFUs seen after 8 days of ctrl or PS_H_ LT-HSC culture (scale bar, 500 μm) (left) and statistical data (right) (*n* = 3 per group). **n** Experimental schema of limiting dilution analysis (LDA) in vivo. **o** Functional HSC frequency determined by LDA at 16 weeks post transplantation (*n* = 6 per group). Dashed lines show 95% confidence intervals. **p** Experimental schema of primary and secondary transplantation (*n* = 5 per group). **q** Donor peripheral-blood chimerism of recipient CD45.1 mice in primary transplantation. **r**, **s** Proportion (**r**) and representative flow cytometry images (**s**) of CD45.2^+^ cells in BM from primary transplantation recipient mice. **t** Absolute number of CD45.2^+^ cells in BM. **u** Donor peripheral-blood chimerism in secondary transplantation. Each symbol represents an individual mouse. Data are shown as the mean ± SD, unpaired two-tailed *t*-test, *P < 0.05, **P < 0.01, ***P < 0.001, n.s., not significant.

We next established mouse models of both short-term (1 week) and long-term (5 weeks) administration of MPs in two different doses, using H_2_O as control (Fig. 1a). The body weight of the three groups of mice did not show any significant discrepancy (Fig. 1b). Strikingly, we observed a notable decrease in white blood cells (WBCs), granulocytes, lymphocytes and monocytes in peripheral blood only from the PS_H_ group in the long-term gavage mice (Fig. 1c–f; Supplementary Fig. S3a–e). We then evaluated the impact of MPs on HSCs, and found that long-term ingestion of MPs markedly decreased both the frequency and absolute number of long-term HSCs (LT-HSCs), which possess the highest self-renewal potential^31^, as well as the multipotent progenitor 3/4 (MPP3/4) cells (Fig. 1g–i). Moreover, apoptotic rates of LT-HSCs, ST-HSCs, MPP2 and MPP3/4 cells were significantly increased in long-term PS_H_ mice (Fig. 1j; Supplementary Fig. S4a, c). Cell cycle analysis revealed that LT-HSCs, ST-HSCs, MPP2 and MPP3/4 cells exhibited a transition from the G_0_ phase into the G_1_ phase in the PS_H_ mice (Fig. 1k, l; Supplementary Fig. S4b, d–f). Additionally, we found that the size and total cell number of colonies were notably smaller in the PS_H_ group, and there was a 23% reduction in colony-forming units (CFUs) relative to the control (Fig. 1m; Supplementary Fig. S4g). In stark contrast, the short-term intake of PS did not have any effect on the number and apoptotic rate of HSCs, as well as their cell cycle status (Supplementary Fig. S3f–o). Together, these findings indicate that MPs dampened the hematopoietic system in both time and dose-dependent manners.

### Long-term ingestion of MPs undermines the reconstitution ability of HSCs

A hallmark manifestation of impaired hematopoietic stem cells is a decline in the ability of reconstitution capacity^32^. In order to test the in vivo repopulation capacity of HSCs, we carried out limiting dilution, competitive repopulation assay (LDA) by transplanting different quantities of donor BM cells (CD45.2) from the ctrl or PS_H_ group, together with recipient BM cells (CD45.1), into mice that had been exposed to lethal levels of radiation (Fig. 1n). Our results showed that the number of competitive repopulating units decreased 4.5-fold in PS_H_ HSCs compared with the ctrl group (Fig. 1o).

Subsequently, we undertook both primary and secondary transplantation assays to assess the long-term repopulation ability of HSCs (Fig. 1p). At 16 weeks post transplantation, we observed a visible reduction in the overall repopulation rate of PS_H_ compared with the ctrl group (Fig. 1q). Moreover, those who received the PS_H_ BM cells showed a significant decrease in both the proportion and absolute number of donor-derived LT-HSCs and MPP2s in BM, as compared to controls (Fig. 1r–t). In secondary transplantation, donor engraftment of the PS_H_ group was much lower compared to the control group (Fig. 1u). We also observed a pronounced reduction in donor-derived T cells and B cells, whereas not the myeloid cells, in both primary and secondary recipient mice (Supplementary Fig. S4h–m). Thus, the long-term consumption of MPs was associated with decreased numbers, high apoptotic rate, abnormal cell cycling, and the reduced ability of graft reconstruction of HSCs.

Furthermore, we assessed whether other MPs with distinct polymer compositions, such as PMMA and PE, had the same impact on the hematopoietic system (Supplementary Fig. S5a). After long-term ingestion in mice, we discovered a visible reduction in WBCs, monocytes and granulocytes of the PE group, and a decrease in WBCs and lymphocytes of the PMMA group (Supplementary Fig. S5b). Similarly, the frequency and absolute number of hematopoietic stem and progenitor cells (HSPCs) were markedly decreased, whereas the apoptotic rate was increased LT-HSCs, in both PMMA and PE intake models (Supplementary Fig. S5c–g). Instead, the cell cycle exit from the G_0_ phase was observed in ST-HSCs after long-term ingestion of PMMA MPs (Supplementary Fig. S5h–k). Overall, our results indicate that long-term ingestion of different types of MPs undermined the self-renewal capacity of HSCs.

### MPs deteriorate HSCs by disrupting gut structure, permeability and microbiota

Although early reports showed that MPs damage hematopoietic homeostasis^20,21^, whether MPs directly or indirectly impact the hematopoietic system is unknown. We cultured the bone marrow cells isolated from C57BL/6J mice with various concentrations of MPs (Supplementary Fig. S6a). Unexpectedly, we found that a very high concentration of MPs did not inhibit cell viability, while even slightly increased total cell number after culture for 7 days (Supplementary Fig. S6b). We treated cultured HSPCs with MPs at 100 μg/mL and 250 μg/mL, and observed constant or increasing frequency and absolute number of HSPCs, together with the decreased apoptotic rate in ST-HSCs, MPP2 and MPP3/4 cells after the treatment of MPs for 14 days (Supplementary Fig. S6c–f). In light of a previous study showing that the mean of the sum quantifiable concentration of plastic particles in human blood was 1.6 µg/mL^17^, we administered MPs intravenously into mouse blood at 0.1 µg/100 µL per week for a duration of 4 weeks to determine the direct impact of MPs on the hematopoietic system (Supplementary Fig. S6g). Our observation revealed that the delivery of MPs into the bloodstream had no impact on the ratio and total count of cells in the blood or HSCs in the bone marrow (Supplementary Fig. S6h–k). Meanwhile, the apoptotic rate, the cell cycle and the ability of HSCs to form colonies (Supplementary Fig. S6l–n) did not alter in response to the intravenous injection of MPs into the mice compared to the control group. These data suggest that long-term in vitro or in vivo exposure to MPs did not damage the hematopoietic system, indicating the effect of MPs in vivo might be due to indirect mechanisms.

**Fig. 2:**
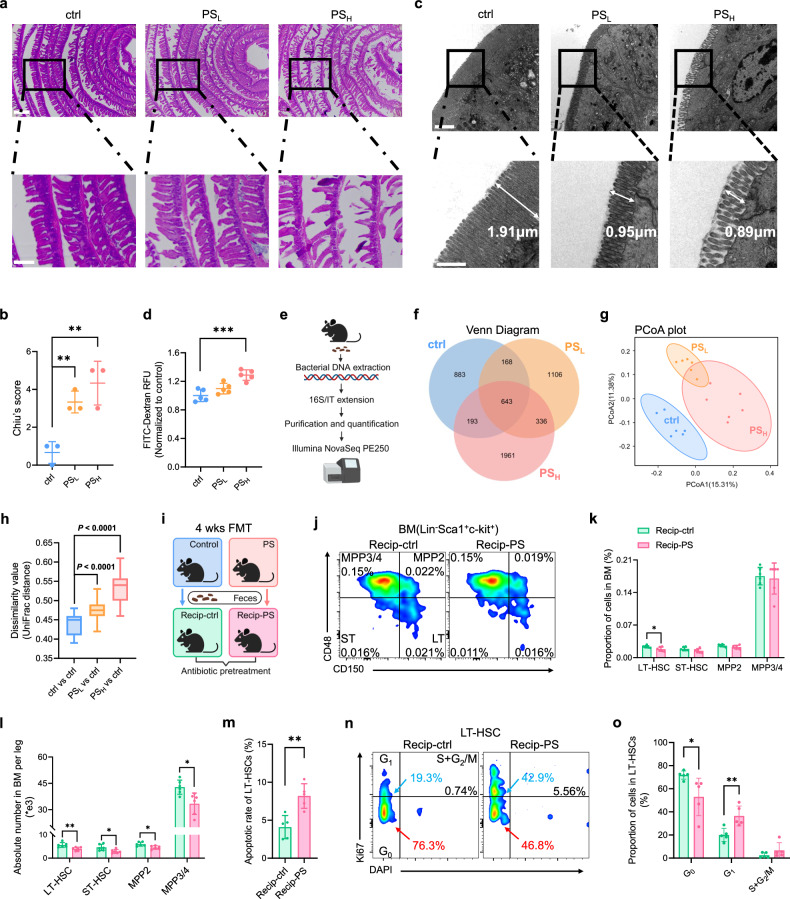
Long-term ingestion of microplastics disrupts gut microbiota and impairs hematopoietic system in mice. **a** Representative images of small intestine stained with hematoxylin-eosin (H&E). The image below (scale bar, 200 μm) is a magnification of the image above (scale bar, 500 μm). **b** Chiu’s score of intestinal section. **c** Representative images of Transmission Electron Microscope (TEM) staining in small intestine. The image below (scale bar, 1 μm) is a magnification of the image above (scale bar, 2 μm). White double headed arrow indicates microvillus length. **d** FITC-dextran uptake in vivo was measured in peripheral blood collected from ctrl, PSL and PSH mice to detect intestinal permeability. **e** Experimental schema of 16s rDNA sequencing from ctrl, PS_L_ and PS_H_ mouse feces (*n* = 6 per group). A detailed list of Operational taxonomic units (OTUs) list is provided in Supplementary Table S1. **f** Venn diagram of three groups. **g**, **h** PcoA plot showing microbial compositional differences between three groups (**g**) as quantified by UniFrac distance (**h**). **i** Schematic of 4-week fecal microbiota transplantation (FMT) (*n* = 6 per group). **j** Representative flow cytometry images. **k**, **l** Proportion (**k**) and absolute number (**l**) of cells in BM from recipient mice. **m** Percentage of apoptotic rate in HSCs. **n**, **o** Representative flow cytometry images of cell cycle (**n**) and proportion within LT-HSCs (**o**) in recipient mice. Error bars indicate SD, unpaired two-tailed *t*-test, *P < 0.05, **P < 0.01, ***P < 0.001, n.s., not significant.

Since we found that MPs were mainly deposited in gastrointestinal tissues (Supplementary Fig. S2b–e), we wondered whether MPs disrupt the gut system. Hematoxylin-eosin (H&E) staining on gut sections revealed that the villous structure of the small intestine was severely disrupted, particularly in the PS_H_ group (Fig. 2a), which was validated by Chiu’s scores^33,34^ (Fig. 2b). Transmission electron microscopy (TEM) analysis of intestinal epithelial cells depicted that the length of microvilli was markedly shortened, and the gap between the microvilli was broadened in the PS_H_ group (Fig. 2c). Moreover, the fluorescein isothiocyanate (FITC)-dextran assay showed increased gut permeability in the PS_H_ group compared with controls (Fig. 2d). These results imply that MPs alter the gut structure and permeability, which is consistent with a previous study^35^.

To determine whether the long-term uptake of MPs altered the intestinal microbiota, we harvested feces from ctrl, PS_L_, and PS_H_ mice for 16 S rDNA sequencing (Fig. 2e; Supplementary Table S1). Notably, we observed that exposure to PS shifted the composition of the microbiota^20,35–37^, as reflected in differential operational taxonomic units (OTUs) between the three groups (Fig. 2f). The principal coordinates (PCoA) and UniFrac dissimilarity distance analyses suggested that the bacterial community in mice that ingested MPs was distinct from that in control mice (Fig. 2g, h). Based on the results above, we hypothesized that changes in the gut caused by MP intake might cause damage to the hematopoietic system. To test this possibility, we undertook fecal microbiota transplantation (FMT) in which wild-type recipient C57BL/6 mice were pretreated with antibiotics and reconstituted with fecal material from the ctrl or PS_H_ mice (Fig. 2i). FMT from the PS_H_ mice resulted in the reduced proportion and absolute number of HSPCs among recipient mice (Fig. 2j–l), as well as increased apoptotic rate and a shorter G_0_ phase in LT-HSCs relative to control mice (Fig. 2m–o; Supplementary Fig. S7). Taken together, these data imply that MPs impact the structure and gut microbiota, leading to the deterioration of the hematopoietic system.

### *Rikenellaceae* and hypoxanthine are responsible for MPs-induced HSC defects

**Fig. 3:**
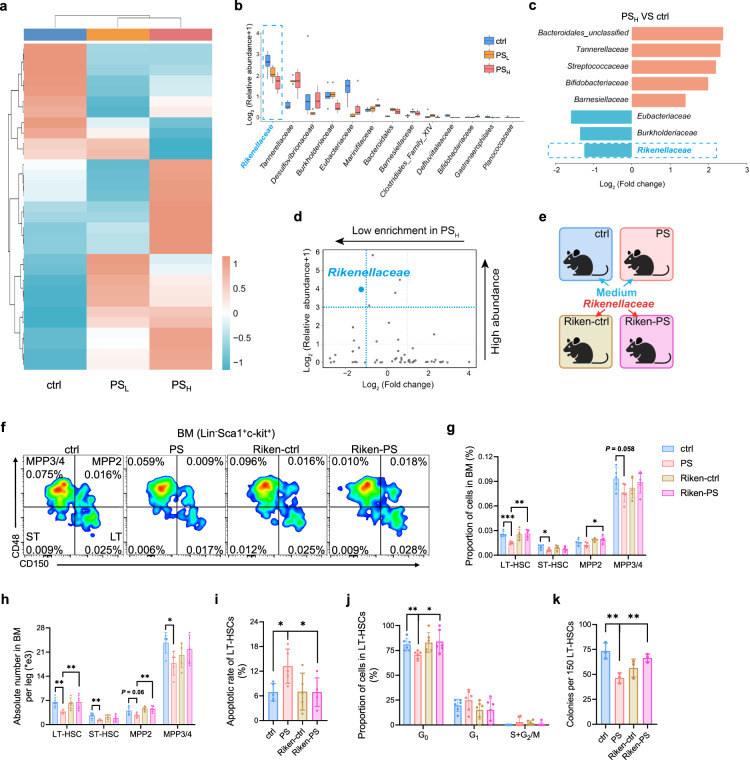
Rikenellaceae is significantly diminished in microplastics intake mice and participates in hematopoiesis. **a**, **b** Heatmap of 16s rDNA sequenced microbial OUT abundances in the ctrl, PS_L_ and PS_H_ group (top 30) (**a**) and histogram of different abundance with significance in three groups (**b**) (*n* = 6 per group). Kruskal test, *P* < 0.05. **c** Statistically significant difference analysis in microbiota from the ctrl and PS_H_ group. Blue bars, enriched in ctrl; red bars, enriched in PS_H_. **d** Volcano plot showing relative abundance distribution of microbial OTUs between ctrl and PS_H_ group. x axis, fold changes in PS_H_ vs ctrl; y axis, relative abundance; blue dot, *Rikenellaceae*. **e** Schematic of *Rikenellaceae* treatment (*n* = 5 per group). **f** Representative flow cytometry images. **g**, **h** Proportion (**g**) and absolute number (**h**) of cells in BM from ctrl, PS, Riken-ctrl and Riken-PS group. **i** Apoptosis levels in LT-HSCs. **j** Cell cycle of LT-HSCs. **k** Statistical plot of CFU from 8 days cultured LT-HSCs (*n* = 3 per group). Error bars indicate SD, unpaired two-tailed *t*-test. *P < 0.05, **P < 0.01, ***P < 0.001.

We next carried out an unbiased clustering analysis of bacterial OTUs (Fig. 3a) to identify which particular bacteria are responsible for the MP-mediated HSC deficits. *Rikenellaceae* was the bacteria with highest abundance after MPs treatment (Fig. 3b). Linear discriminant analysis (LDA) showed that five taxa (*Bacteroidales*, etc.) were enriched, whereas three taxa (*Rikenellaceae*, *Burkholderiaceae*, *Eubacteriaceae*) were reduced in the PS_H_ mice compared with controls (Fig. 3c). A volcano plot flagged *Rikenellaceae* as the most reduced and represented bacteria in the PS_H_ mice (Fig. 3d). These results evoked us to hypothesize that *Rikenellaceae*, the most abundant bacteria that was reduced in the gut flora of MP-treated mice, might have a protective effect on the hematopoietic system, as reported previously^29^.

To test this, we inoculated the ctrl and PS_H_ group mice with *Rikenellaceae* by oral gavage three times a week for 4 weeks (Fig. 3e), using bacterial growth medium (Reinforced Clostridial Medium) as a control. Remarkably, the PS_H_ mice inoculated with *Rikenellaceae* exhibited a significant recovery in both the ratio and quantity of HSCs (Fig. 3f–h). The infusion of *Rikenellaceae* substantially reverted the rise in apoptotic rate and the drop in the G_0_ phase of LT-HSCs caused by MPs (Fig. 3i, j; Supplementary Fig. S8a–f). Finally, we sorted HSCs from the four groups and cultured them ex vivo for 8 days. The reduced number and size of colonies by MPs were rescued by *Rikenellaceae* treatment (Fig. 3k; Supplementary Fig. S8g).

**Fig. 4:**
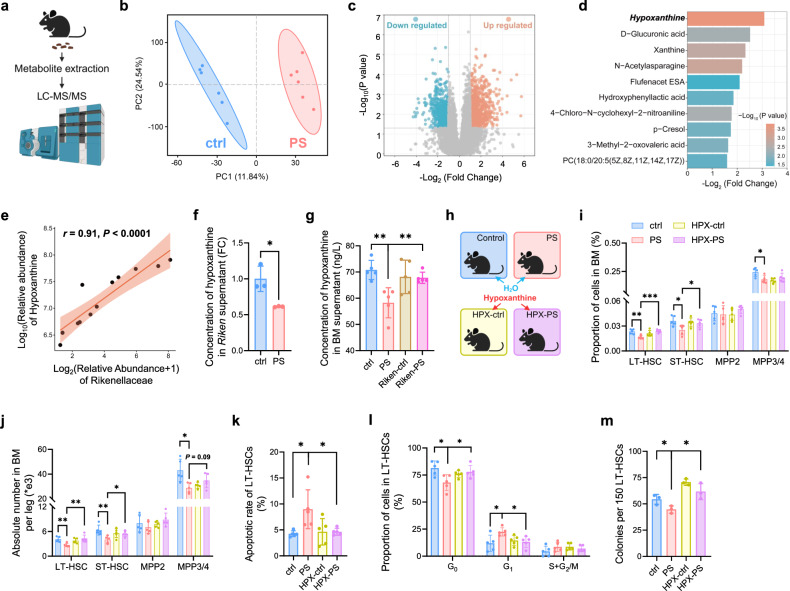
Administration of hypoxanthine ameliorates the effect of MPs on HSCs. **a** Schematic overview of metabolome sequencing from ctrl and PS_H_ mice feces (*n* = 6 per group). **b** PCA plot showed compositional differences of metabolites between ctrl and PS_H_ group. **c** Volcano plot of differentially metabolites in two groups. Blue dot, downregulated in PS_H_; red dot, upregulated in PS_H_ group. A detailed metabolites taxa list is provided in Supplementary Table S2. **d** Metabolite set enrichment analysis identified metabolites decreased the most in PS_H_ group. **e** Pearson’s correlation plot of *Rikenellaceae* and hypoxanthine in ctrl or PS_H_ mice feces (*n* = 6 per group). *r*, Pearson correlation coefficient; *P* < 0.0001. **f** Concentration of hypoxanthine in *Rikenellaceae* supernatant (*n* = 3 per group). **g** Concentration of hypoxanthine in BM supernatant of *Rikenellaceae* treated group. **h** Schematic of hypoxanthine metabolite treatment (*n* = 5 per group). Proportion (**i**) and absolute number (**j**) of cells in BM from ctrl, PS, HPX-ctrl and HPX-PS group. **k** Apoptotic rate of LT-HSCs. **l** Cell cycle of LT-HSCs. m Numbers of CFU from 8 days cultured LT-HSCs (*n* = 3 per group). Error bars indicate SD, unpaired two-tailed *t*-test. *P < 0.05, **P < 0.01, ***P < 0.001.

Given the metabolite alterations in response to MPs^20,21,38^, we next performed untargeted metabolomics analysis in fecal samples from the ctrl and PS_H_ group to assess which metabolites mediated the deleterious roles of MPs in HSCs (Fig. 4a; Supplementary Table S2). Principal component analysis (PCA) and volcano plots displayed distinct metabolite profiles between the PS_H_ and the ctrl group (Fig. 4b, c), among which the purine derivatives, hypoxanthine and xanthine, showed the most significant decrease after long-term gavage of MPs in the PS_H_ group relative to the ctrl group (Fig. 4d). Besides, we found a positive correlation between the relative abundance of *Rikenellaceae* and hypoxanthine (Fig. 4e), concordant with our 16s rDNA sequencing data (Fig. 3b–d) and previous studies^39,40^. In addition, we revealed that the presence of PS caused a decrease in the concentration of hypoxanthine both in the supernatant of *Rikenellaceae* and bone marrow (Fig. 4f, g). However, this drop in the bone marrow was effectively reversed after *Rikenellaceae* treatment in the PS_H_ mice (Fig. 4g), implying that hypoxanthine was likely a product of *Rikenellaceae* in the gut.

Following the metabolite assessment, we treated C57BL/6 mice with PS MPs for 5 weeks before feeding them with hypoxanthine-supplemented water (Fig. 4h). Flow cytometry revealed that both the frequency and absolute number of HSPCs in bone marrow were rescued in PS_H_ mice with hypoxanthine treatment (Fig. 4i, j), accompanied with the abrogation of diminished apoptotic rate and increased quiescence state (Fig. 4k, l; Supplementary Fig. S9a–f). Meanwhile, we sorted HSCs from bone marrow in the ctrl, PS, HPX-ctrl and HPX-PS groups and measured the colony formation ability. The reduced number and size of colonies by MPs were fully rescued by the administration of hypoxanthine (Fig. 4m; Supplementary Fig. S9g). Additionally, we treated BM cells from mice with 100 pg/mL hypoxanthine for 3 days and observed significant increases in both the ratio and absolute number of HSCs, along with a reduction in apoptosis and an enhanced capacity to form colonies in vitro (Supplementary Fig. S9h–n). In sum, our studies suggest that the administration of *Rikenellaceae* or hypoxanthine could ameliorate the adverse effects of MPs on HSCs.

### Long-term ingestion of MPs inactivates HPRT-Wnt signaling in HSCs

**Fig. 5:**
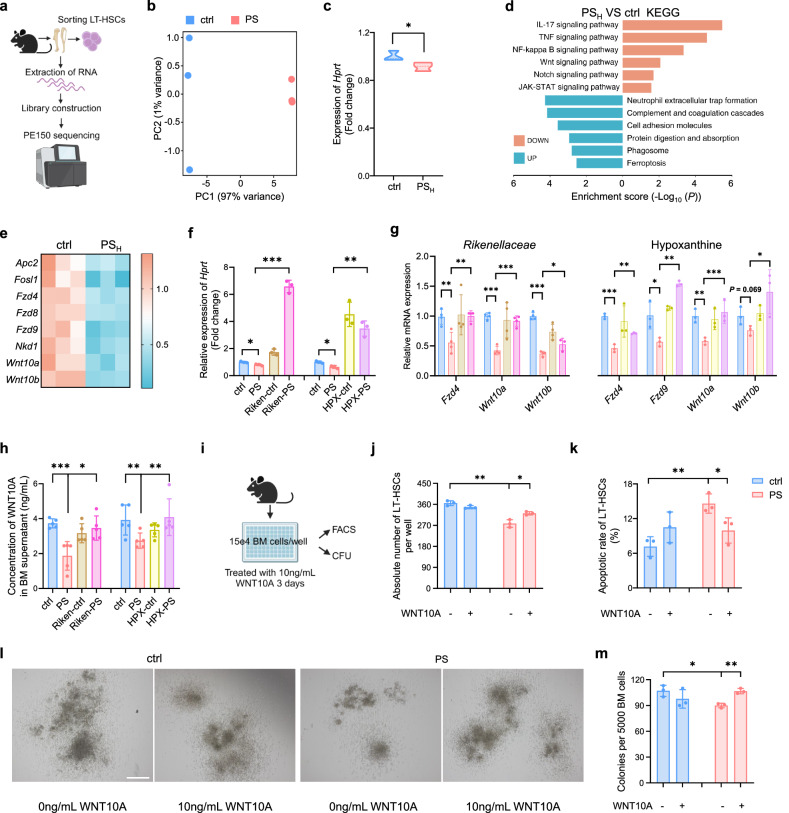
HSCs defects in microplastic model are associated with defects in the Wnt signaling pathway. **a** Schematic of RNA-seq workflow (*n* = 3 per group). **b** PCA plot of BM LT-HSCs in ctrl and PS_H_ group. **c** Expression of *Hprt* in LT-HSCs. **d** KEGG analysis of upregulated and downregulated signaling pathways in PS_H_ mice. Red bars, downregulated in PS_H_; blue bars, upregulated in PS_H_. **e** Relative expression of genes in the Wnt signaling pathway. **f** Relative expression of *Hprt* in *Rikenellaceae* treatment or hypoxanthine treatment group (*n* = 3 per group). **g** Relative mRNA expression of genes within *Rikenellaceae* treatment group or hypoxanthine treatment group. **h** Enzyme-linked immunosorbent assay (ELISA) to detect WNT10A in BM supernatant within *Rikenellaceae* treatment group or hypoxanthine treatment group (*n* = 5 per group). **i** Schematic outlining BM cell analysis (*n* = 3 per group). **j** Absolute number of LT-HSCs. **k** Apoptosis levels in LT-HSCs. **l**, **m** Representative images of CFUs from 8-day cultured BM cells (scale bar, 500 μm) (**l**) and statistical data (**m**). Error bars indicate SD, unpaired two-tailed *t*-test. *P < 0.05, **P < 0.01, ***P < 0.001.

To further dissect the potential downstream mechanisms accounting for HSC defects after long-term gavage of MPs, we sorted LT-HSCs from the ctrl and PS_H_ mice and conducted RNA sequencing (RNA-seq) to identify transcriptomic changes (Fig. 5a; Supplementary Table S3). Of note, the PCA plot showed a significant variation between the PS_H_ group and ctrl group (Fig. 5b). In parallel with the lower levels of hypoxanthine (Fig. 4d), we observed a minor reduction in the expression of hypoxanthine-guanine phosphoribosyltransferase (HPRT) (Fig. 5c), an enzyme that catalyzes the conversion of hypoxanthine and guanine to create purine nucleotides. Partial deficiency of hypoxanthine and HPRT suggested that the purine salvage pathway was compromised, which has previously been linked to neural diseases, malignant tumors and dysfunction of HSCs^41,42^.

Kyoto Encyclopedia of Genes and Genomes (KEGG) analysis identified the “Wnt signaling pathway” as one of the most enriched down-regulated pathways in the PS-treated HSCs (Fig. 5d), consistent with previous studies that deletion of HPRT coordinately dysregulates the Wnt signaling pathway^43^. The canonical Wnt cascade has also been shown as a crucial regulator of HSCs^44^. Besides, gene set enrichment analysis (GSEA) suggested that the Wnt receptor signaling pathway was down-regulated in LT-HSCs from the PS_H_ group compared to the ctrl group (Supplementary Fig. S10a). Indeed, the mRNA levels of Wnt signaling pathway-associated genes, such as *Fzd4*, *Wnt10a* and *Wnt10b*, were markedly reduced after exposure to MPs (Fig. 5e). We next determined the expression of Wnt signaling related genes by quantitative RT-PCR and enzyme-linked immunosorbent assay (ELISA), and found that the declined expression of *Hprt* and Wnt signaling genes, such as *Wnt10a*, was significantly restored after administration with *Rikenellaceae* or hypoxanthine (Fig. 5f–h). Moreover, adding hypoxanthine to BM cells significantly enhanced the expression of genes associated with the Wnt signaling pathway, at both the protein and RNA levels (Supplementary Fig. S9o, p). To characterize the functional context of WNT10A in HSCs, we cultured bone marrow cells from controls and PS-treated mice and supplemented with or without WNT10A in the medium (Fig. 5i). After 3 days of culture, we observed that WNT10A treatment markedly increased the absolute number, decreased apoptotic rate and increased colonies of HSCs in the PS_H_ group (Fig. 5j–m; Supplementary Fig. S10b–d). Given the observed downregulation of IL-17, TNF, and NF-kappa B signaling pathways after prolonged exposure to MPs (Fig. 5d; Supplementary Fig. S10e), we administered IL-17, TNF, or NF-kappa B to bone marrow cells from the mice that had been treated with MPs. Nevertheless, we noticed that these interventions were unable to rectify the deficiency of HSCs, including their overall cell number, cell death and capacity to generate colonies (Supplementary Fig. S10f–i). Collectively, these data hint that inactivation in the HPRT-Wnt cascade, rather than in the IL-17, TNF, or NF-kappa B signaling pathway, was indispensable for hematopoietic damage after MP intake.

### High exposure to microplastics in healthy donors correlates with engraftment failure in patients receiving allogeneic HSCT

**Fig. 6:**
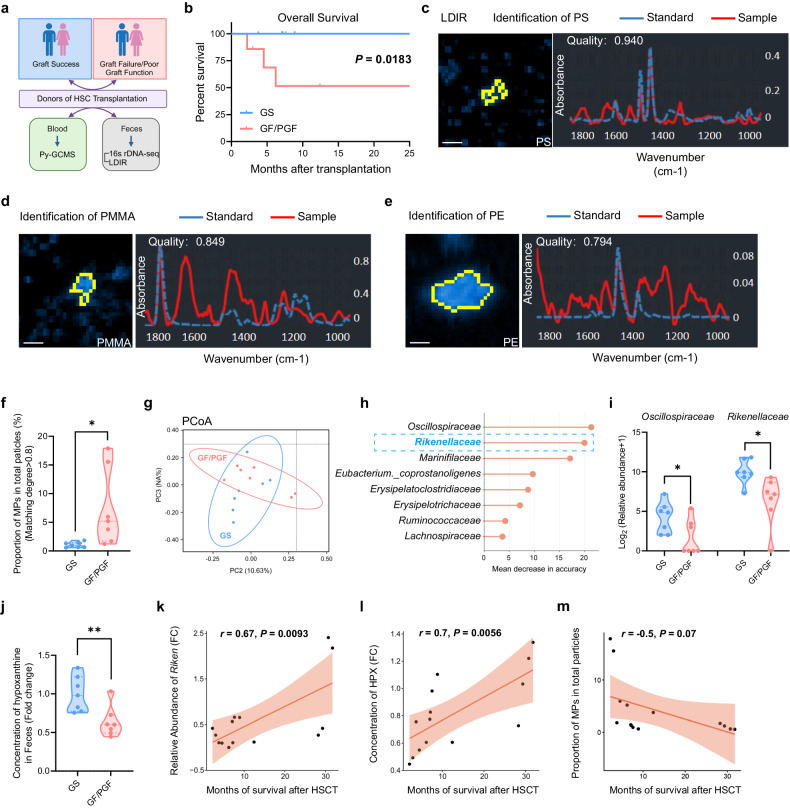
Microplastics and gut microbiota in donors are significantly associated with outcome of HSCT in patients. **a** Scheme of the method to collect patient samples and detect relevant indicators (graft success (GS): 7 patients; graft failure/poor graft function (GF/PGF): 7 patients). **b** Survival rate of patients after BM transplantation. **c**–**e** Representative images of MPs in feces through laser direct infrared imaging (LDIR). Picture (left), definition curve (right); identification of PS (**c**), PMMA (**d**) and PE (**e**). Scale bars, 30 μm. **f** Relative quantification of MPs in feces. **g** PCoA of 16s rDNA in feces. **h** Random Forest (RF) analysis of feces in two groups. **i** Relative abundance of *Oscillospiraceae* and *Rikenellaceae* in feces. **j** Concentration of hypoxanthine in feces. **k**–**m** Correlation between survival rate after BMT and relative abundance of *Rikenellaceae* (**k**), concentration of hypoxanthine (**l**) or proportion of MPs in feces (**m**). The Pearson correlation coefficient (*r*) and empirical *P* value are also shown. Error bars indicate SD, unpaired two-tailed *t*-test. *P < 0.05, **P < 0.01.

To determine whether the relationship among MPs, the microbiota, and metabolites holds true in humans, we conducted a double-blind clinical study. Fecal and blood samples were collected from 14 healthy donors whose CD34^+^ HSPCs have been transplanted into patients with acute myelocytic leukemia (AML), acute lymphocytic leukemia (ALL) or myelodysplastic syndromes (MDS) (Fig. 6a; Supplementary Table S4). Based on the engraftment status of patients after HSCT, patients were classified into two groups, graft success (GS) and graft failure or with poor graft function (GF/PGF), which showed a massive difference in the survival time of patients (Fig. 6b). By carrying out laser direct infrared imaging (LDIR), we detected microplastics of PS, PMMA and PE in the donors’ feces samples, and found a higher proportion of microplastics among all detected MPs in GF/PGF donors compared to GS donors (Fig. 6c–f). Furthermore, by performing pyrolysis gas chromatography/mass spectrum analysis (Py-GC/MS), we identified multiple types of MPs in the donors’ blood samples, including PS and PET, and revealed higher levels of MPs in the blood of GF/PGF donors compared to GS group (Supplementary Fig. S10j).

We next profiled bacterial 16S rDNA genes in the feces of GS and GF/PGF donors (Supplementary Table S5). PCoA showed that microbiome composition was distinct between GF/PGF and GS donor samples (Fig. 6g). To analyze which gut microbiota played a pivotal role, we executed the Random Forest analysis and showed that *Oscillospiraceae* and *Rikenellaceae* were the most significant bacteria in donors’ feces (Fig. 6h). Concordantly, the abundance of *Rikenellaceae* and *Oscillospiraceae* was profoundly reduced in the feces of GF/PGF donors (Fig. 6i; Supplementary Fig. S10k). Meanwhile, the level of hypoxanthine in the feces was also decreased in the GF/PGF donors (Fig. 6j). Finally, we calculated the Pearson correlation coefficients between the survival time of patients and the levels of microplastics, *Rikenellaceae* or hypoxanthine, and observed strong positive correlations between the survival of patients and the abundance of *Rikenellaceae* (Pearson’s *r* = 0.67) or hypoxanthine (Pearson’s *r* = 0.7), as well as negative correlation between the survival of patients and MP levels in feces (Pearson’s *r* = −0.5) (Fig. 6k–m). Moreover, the levels of MPs, *Rikenellaceae*, and hypoxanthine were closely related (Supplementary Fig. S10l–n). To sum up, the link between MPs, *Rikenellaceae* and hypoxanthine uncovered in mice also held true in human donors for HSCT and correlated with graft success.

## Discussion

In this study, we comprehensively demonstrate the deleterious effect of long-term exposure to MPs on the self-renewal of the HSCs both in vitro and in vivo. The impact of MPs on HSCs is both time- and dose-dependent, given that ingesting MPs for short periods or in low concentrations does not impair the hematopoietic system. Mechanistically, MPs dampen the self-renewal and reconstitution abilities of HSCs by altering the gut *Rikenellaceae*-hypoxanthine-HPRT-Wnt signaling axis (Supplementary Fig. S10o). We reveal that administration of *Rikenellaceae* or hypoxanthine in mice, as well as treatment of WNT10A in the culture system, effectively protect HSC from MPs-induced damage. Additionally, we validate our findings in human patients receiving allogenic HSCT, whose survival time is negatively correlated with levels of MPs, while positively with the abundance of *Rikenellaceae*, and hypoxanthine in the HSC donors’ feces and blood. Collectively, our study unleashes the detrimental roles and precise mechanisms of MPs in HSCs, which might provide potential strategies to prevent hematopoietic damage from MPs and serve as a fundamental critique for selecting suitable donors for HSCT in clinical practice.

To date, numerous studies have demonstrated that MPs with different sizes cause damage to multiple organs in mice by a similar mechanism that MPs modify the gut microbiota composition^18,20,21,35–37^. Oral exposure to 0.5 and 50 μm polystyrene MPs induced hepatic lipid disorder^37^. 5 μm polystyrene MPs caused intestinal barrier dysfunction and metabolic disorders^35^. 10–150 μm polyethylene MPs generated obvious inflammation in the intestine^36^. Furthermore, nanoplastics (NPs) (< 100 nm) could pass through barriers and enter the bone marrow in a previous study^45^, and nanoplastics were found in the bone marrow and disrupted hematopoietic homeostasis^20,21^, suggesting that the impact of nanoplastics might act directly in mouse bone marrow. By contrast, our data demonstrate MPs (500 nm) were detected in the gastrointestinal tract and peripheral blood, and dampened the self-renewal of HSCs by disrupting the gut microbiota and metabolites. Moreover, we found that MPs neither showed a negative effect when co-cultured directly with HSCs, nor had an adverse impact on the hematopoietic system after tail vein injection into the blood of mice. These contradictory results might result from the different sizes of MPs, with the smaller NPs penetrating into bone marrow while the larger MPs damaging HSCs by altering the gut microbiota. The comprehensive comparison of the same conditions between MPs and NPs should be studied in the future.

Previous studies report that the concentration of hypoxanthine is positively correlated with the level of *Rikenellaceae*^39,40^. Consistently, we found that the content of *Rikenellaceae* in the gut microbiome and hypoxanthine in the plasma decreased simultaneously in mice oral gavage with MPs, and MPs-treated *Rikenellaceae* reduced the level of hypoxanthine, reinforcing that hypoxanthine might be a product of *Rikenellaceae*. Furthermore, we identified the HPRT and the Wnt signaling pathway as the downstream mechanisms underlying the effects of MPs on HSCs. Indeed, a previous study reveals that HSPCs rely on HPRT-associated purine salvaging, as lower HPRT activity leads to severely reduced competitive repopulation ability and altered cell-cycle progression^42^. However, it is unclear about the precise roles of HPRT-Wnt in HSCs. Here, we offer a new perspective that the hypoxanthine-HPRT-Wnt signaling pathway plays a crucial role in HSCs, given that HPRT deficiency and abnormal hypoxanthine are accompanied by aberrations in Wnt/β-catenin signaling pathway in early neuro-developmental processes and neurodegenerative disease^43,46^. Meanwhile, current evidence emerges that the Wnt cascade is one of the most essential regulators in controlling stem cell self-renewal^44,47,48^. These results consolidate our hypothesis that long-term exposure to MPs diminishes the content of *Rikenellaceae* and hypoxanthine in the gut, the latter of which circulates through the peripheral blood, inactivates the HPRT and Wnt signaling, and eventually dampens the self-renewal and reconstitution capabilities of HSCs in the bone marrow. Our work highlights the gut-bone marrow axis as a new regulatory route for how environmental contaminants influence the hematopoietic system.

While alterations in bacterial species and their metabolites resulting from MPs or NPs are known to be associated with the impaired hematopoietic system in mice^20,21^, there is a lack of sufficient prospective clinical data on the role of MPs in microbiome and metabolites of humans and whether they can affect the human hematopoietic system. We note that the content of MPs ingested by donors of HSCT patients also influences the outcome of transplantation. Consuming more MPs reduces the level of *Rikenellaceae* and hypoxanthine, as seen in the mouse model, resulting in functional abnormalities in HSCs and a worse transplant success rate in humans. In our study, we highlight the conservation of MPs-induced HSC damage in humans and mice, which proves the gut-bone marrow axis as a new regulatory route for how environmental contaminants influence the hematopoietic system, and indicates that gut microbiota and metabolites could be potentially used as clinical selection criteria for donors of patients with HSCT.

## Methods

### Mice

C57BL/6J (CD45.2) and C57BL6.SJL (CD45.1) mice were purchased from The Jackson Laboratory and housed under specific pathogen-free conditions. Male and female mice from 8 to 12 weeks were used in experiments and provided with a suitable environment and sufficient water and food. After a week of acclimatization, each mouse was randomly divided into groups, given 100 μL pure water, 0.01 mg/100 μL, or 0.1 mg/100 μL MPs by oral gavage every two days for five weeks in a gavage experiment (*n* = 5 for each group). For the intravenous injection experiment, MPs were administered into mouse blood via the tail vein at a rate of 0.1 µg/100 µL per week for a duration of 4 weeks (*n* = 5 for each group). All animal experiments were first approved by the Laboratory Animal Welfare and Ethics Committee of Zhejiang University (AP CODE: ZJU20220108).

### Microplastics characterization

Indocyanine green polystyrene (ICG-PS), polystyrene (PS) and polymethyl methacrylate (PMMA) particles were obtained from Suzhou Mylife Advanced Material Technology Company (China). Polyethylene (PE) particles were purchased from Cospheric (USA). Scanning electron microscopy (SEM, Nova Nano 450, FEI) was used to characterize the primary sizes and shapes of different MPs^20^. MPs were dispersed in ultrapure water with sonication before dynamic light scattering analysis (Zetasizer, Malvern, UK) to determine the hydrodynamic sizes and zeta potentials^49^.

### Imaging of organs ex vivo

Mice were sacrificed and organs were removed within six hours of ICG-PS gavage, including the heart, lung, kidney, spleen, liver, gastrointestinal tissues and bone marrow. Feces were collected 1 h before the mice were sacrificed. Both organs and feces were monitored by ex vivo bioluminescence imaging with a small-animal imaging system^50^ (IVIS Spectrum, PerkinElmer).

### Flow cytometry and cell sorting

For flow cytometry analysis and isolation of hematopoietic stem and progenitor cells, cells were stained with relevant antibodies^51^ in PBS with 2% fetal bovine serum for 30−45 min on ice. Antibody clones that were used: Sca-1-PE-Cy7, c-Kit-APC, CD150-PE, CD48-BV421, CD45.1-FITC, CD45.2 PE-Cy5, Gr-1-PE-Cy5, Mac1-PE-Cy5, IgM-PE-Cy5, CD3-PE-Cy5, CD4- PE-Cy5, CD8-PE-Cy5, CD45R-PE-Cy5 and Ter-119-PE-Cy5. Detailed antibody information is summarized in Supplementary Table S6. HSPCs were stained with a lineage antibody cocktail (Gr-1, Mac1, CD3, CD4, CD8, CD45R, TER119 and B220), Sca-1, c-Kit, CD150 and CD48. Cell types were defined as followed: LSK compartment (Lin^−^Sca-1^+^c-Kit^+^), LT-HSC (LSK CD150^+^CD48^−^), ST-HSC (LSK CD150^−^CD48^−^), MPP2 (LSK CD150^+^CD48^+^) and MPP3/4 (LSK CD150^−^CD48^+^). B cells (CD45.2^+^Mac1^−^Gr-1^+^B220^+^), T cells (CD45.2^+^Mac1^−^Gr-1^+^CD3^+^) and myeloid cells (CD45.2^+^Mac1^+^Gr-1^−^). Samples were analyzed on a flow cytometer (CytoFLEX LX, Beckman). For sorting HSCs, lineage antibody cocktail-conjugated paramagnetic microbeads and MACS separation columns (Miltenyi Biotec) were used to enrich Lin^−^ cells before sorting. Stained cells were re-suspended in PBS with 2% FBS and sorted directly using the Beckman moflo Astrios EQ (Beckman). Flow cytometry data were analyzed by FlowJo (BD) software.

### Apoptosis and cell cycle assays

Apoptosis of cells was detected by Annexin V staining (Yeason, China). After being extracted from the bone marrow of mice, 5 × 10^6^ cells were labeled with different surface markers for 30 to 45 min at 4 °C and then twice rinsed with PBS. Subsequently, the cells were reconstituted in binding buffer and supplemented with Annexin V. After 30 min of incubation, flow cytometry was detected in the FITC channel. Cell cycle analysis was performed with the fluorescein Ki-67 set (BD Pharmingen, USA), following the directions provided by the manufacturer. Briefly, a total of 5 × 10^6^ bone marrow cells were labeled with corresponding antibodies, as previously stated. Afterward, the cells were pre-treated with a fixation/permeabilization concentrate (Invitrogen, USA) at 4 °C overnight and subsequently rinsed with the binding buffer. The cells were stained with Ki-67 antibody for 1 h in the dark and then with DAPI (Invitrogen) for another 5 min at room temperature. Flow cytometry data were collected by a flow cytometer (CytoFLEX LX, Beckman, USA).

### In vitro colony-forming unit (CFU) assay

HSCs were sorted by flow cytometry according to the experimental group (ctrl and PS_H_ mice, *Rikenellaceae* treatment or hypoxanthine treatment). 150 HSCs were seeded in triplicate on methylcellulose media^52^ (M3434, Stemcell Technologies, Inc.). After 8 days, the number of colonies was counted by microscopy. In addition, 5000 BM cells were seeded and analyzed the same way as HSCs. The cell culture media was diluted in PBS and subjected to centrifugation at 400× *g* for 5 min to determine the total cell number.

### Competitive transplantation assay

Recipient mice (CD45.1) were administered drinking water with Baytril (250 mg/L) for 7 days pre-transplant and 10 days post-transplant. The day before transplantation, recipients received a lethal dose of radiation (4.5 Gy at a time, divided into two times with an interval of 4 h). In primary transplantation, 2 × 10^5^ bone marrow cells from the ctrl or PS group (CD45.2) mice and 2 × 10^5^ recipient-type (CD45.1) bone marrow cells were transplanted into recipient mice (CD45.1) mice. Cells were injected into recipients via tail vein injection. Donor chimerism was tracked using peripheral blood cells every 4 weeks for at least 16 weeks after transplantation. For secondary transplantation, donor BM cells were collected from primary transplant recipients sacrificed at 16 weeks after transplantation and transplanted at a dosage of 2 × 10^6^ cells into irradiated secondary recipient mice (9 Gy). Analysis of donor chimerism and the cycle of transplantation in secondary transplantation were the same as in primary transplantation.

### Limiting dilution assays

For limiting dilution assays^52^, 1 × 10^4^, 5 × 10^4^ and 2 × 10^5^ donor-derived bone marrow cells were collected from ctrl or PS mice (CD45.2) and transplanted into irradiated (9 Gy) CD45.1 recipient mice with 2 × 10^5^ recipient-type (CD45.1) bone-marrow cells. Limiting dilution analysis was performed using ELDA software^53^. 16 weeks after transplantation, recipient mice with more than 1% peripheral-blood multilineage chimerism were defined as positive engraftment. On the other hand, recipient mice undergoing transplantation that had died before 16 weeks post transplantation were likewise evaluated as having failed engraftment^54^.

### Histological analysis and TEM of small intestines

For histological analysis, small intestines were collected and fixed in 4% paraformaldehyde and embedded in paraffin, sectioned (5 μm thickness), and stained with H&E at ZJU Animal Histopathology Core Facility (China). We used Chiu’s scores^33,34^ to evaluate the damage for each sample. The grade was as follows: 0, normal mucosa; 1, development of subepithelial Gruenhagen’s space at the tip of villus; 2, extension of the Gruenhagen’s area with moderate epithelial lifting; 3, large epithelial bulge with a few denuded villi; 4, denuded villi with lamina propria and exposed capillaries; and 5, disintegration of the lamina propria, ulceration, and hemorrhage. For TEM analysis, slices of the small intestine were fixed with 2.5% glutaraldehyde for ultra-microstructure observation of intestinal epithelial cells. The samples were postfixed for one hour at 4 °C with 1% osmium tetroxide and 30 min with 2% uranyl acetate, followed by dehydration with a graded series of alcohol solutions (50%, 70%, 90% and 100% for 15 min each) and acetone (100% twice for 20 min). Subsequently, they were embedded with epon (Sigma-Aldrich, MO, US) and polymerized. Ultrathin sections (60−80 nm) were made, and examined using TEM (Tecnai G2 Spirit 120 kV, Thermo FEI).

### Intestine permeability assay

In the short-term and long-term mouse models for MP ingestion, mice were fasted for 4 h before oral gavage of FITC-dextran (4 kD, Sigma). The fluorescence intensity of FITC-dextran (50 mg/100 g body weight) was measured in the peripheral blood after 2 h of gavage. Fluorescence was measured using a microplate reader (Molecular Devices, SpectraMax iD5) with excitation at 490 nm and emission at 520 nm^29^.

### Fecal 16S rDNA sequencing

Fecal samples (about 30−50 mg per sample) were collected from the ctrl, PS_L_ and PS_H_ mice, quickly frozen in liquid nitrogen, and stored at −80 °C. DNA samples for the microbial community were extracted using E.Z.N.A.® Stool DNA Kit (Omega, USA), according to the manufacturer’s instructions. In brief, polymerase chain reaction (PCR) amplification of prokaryotic 16S rDNA gene V3–V4 region was performed using the forward primer 341 F (5’-CCTACGGGNGGCWGCAG-3’) and the reverse primer 805 R (5’-GACTACHVGGGTATCTAATCC-3’)^55^. After 35 cycles of PCR, sequencing adapters and barcodes were included to facilitate amplification. The PCR products were detected by 1.5% agarose gel electrophoresis and were further purified using AMPure XT beads (Beckman Coulter Genomics, Danvers, MA, USA), while the target fragments were recovered using the AxyPrep PCR Cleanup Kit (Axygen, USA). In addition, the amplicon library was quantified with the Library Quantification Kit for Illumina (Kapa Biosciences, Woburn, MA, USA), and sequenced on the Illumina NovaSeq PE250 platform. In bioinformatics pipeline^29,56^, the assignment of paired-end reads to samples was determined by their unique barcode, and subsequently shortened by cutting off the barcode and primer sequence. The paired-end reads were combined by FLASH (v1.2.8). Quality filtering on the raw reads was carried out under precise parameters to obtain high-quality clean tags according to fqtrim (v0.94). The chimeric sequences were filtered by Vsearch software (v2.3.4). After the dereplication process using DADA2, we acquired a feature table and feature sequence. The bacterial sequence fragments obtained were grouped into Operational Taxonomic Units (OTUs) and compared to the Greengenes microbial gene database using QIIME2. Alpha diversity and beta diversity were generated by QIIME2, and pictures were drawn by R (v3.2.0). The species annotation sequence alignment was performed by Blast, with the SILVA and NT-16S databases as the alignment references. Additional sequencing results are provided in Supplementary Table S1. The experiment was supported by Lc-Bio Technologies.

The methods for the analysis of feces from HSCT donors were slightly different from those used for mice. All samples were stored in the GUHE Flora Storage buffer (GUHE Laboratories, China). The bacterial genomic DNA was extracted with the GHFDE100 DNA isolation kit (GUHE Laboratories, China) and quantified using a NanoDrop ND-1000 spectrophotometer (Thermo Fisher Scientific, USA). The V4 region of the bacterial 16S rDNA genes was amplified by PCR, with the forward primer 515 F (5’-GTGCCAGCMGCCGCGGTAA-3’) and the reverse primer 806 R (5’-GGACTACHVGGGTWTCTAAT-3’). PCR amplicons were purified with Agencourt AMPure XP Beads (Beckman Coulter, IN) and quantified by the PicoGreen dsDNA Assay Kit (Invitrogen, USA). Following the previously reported steps^57^, the paired-end 2 × 150 bp sequencing was performed on the Illumina NovaSeq6000 platform. The details of bacterial OTUs are summarized in Supplementary Table S5. Sequence data analyses were performed using QIIME2 and R packages (v3.2.0).

### Metabolomic analysis

For metabolite evaluation, samples from mice feces were prepared and detected as previously described^55,58,59^. In a nutshell, metabolites were extracted from feces through precooled 50% methanol buffer and stored at −80 °C before the LC‐MS analysis. All chromatographic separations were conducted using an ultra-performance liquid chromatography (UPLC) system (SCIEX, UK). A reversed phase separation was performed using an ACQUITY UPLC T3 column (100 mm * 2.1 mm, 1.8 µm, Waters, UK). The temperature of the column oven was maintained at 35 °C and the flow rate was 0.4 mL/min. Both positive (the ionspray voltage floating set at 5000 V) and negative ion modes (−4500 V) were analyzed using a TripleTOF 5600 Plus high-resolution tandem mass spectrometer (SCIEX, UK). The mass spectrometry data were obtained in Interactive Disassembler Professional (IDA) mode, with a time-of-flight (TOF) mass range of 60 to 1200 Da. The survey scans were acquired in 150 milliseconds and product ion scans with a charge state of 1+ and 100 counts per second (counts/s) were recorded up to 12. Cycle duration was 0.56 s. Stringent quality assurance (QA) and quality control (QC) procedures were applied, as the mass accuracy was calibrated every 20 samples and a QC sample was obtained every 10 samples. LCMS raw data files underwent processing in XCMS (Scripps, La Jolla, CA) to perform peak picking, peak alignment, gap filling, and sample normalization. Online KEGG was adopted to annotate metabolites through the matching between the precise molecular mass data (m/z) of samples and those from the database. PCA and volcano plot were utilized to identify ion characteristics that exhibit significant differences between the groups. The details of metabolomes can be found in Supplementary Table S2. The experiment was supported by Lc-Bio Technologies.

### Fecal microbiota transplantation

Before FMT, SPF mice received a 200 μL antibiotic treatment (1 g/L ampicillin, 0.5 g/L neomycin, 0.5 g/L vancomycin and 1 g/L metronidazole) for three consecutive days by oral gavage. Fresh feces were collected from ctrl or PS mice and resuspended in reduced PBS (0.5 g/L cysteine and 0.2 g/L Na_2_S in PBS) at a ratio of about 120 mg feces/mL reduced PBS. Feces were then centrifuged at 500× *g* for 1 min to remove insolubilize particles^25^. Recipients (C57BL/6 J mice) were administered 100 mL of the supernatant from different groups by oral gavage twice every week for 4 weeks. 2 days after the last FMT, recipients were euthanized to analyze the changes in the hematopoietic system.

### *Rikenellaceae* and hypoxanthine treatment

The *Rikenellaceae* strain (ATCC BAA-1961), purchased from ATCC, was cultured in an anaerobic chamber using BD Difco™ Dehydrated Culture Media: Reinforced Clostridial Medium at a temperature of 37 °C with a gas mixture of 80% N_2_ and 20% CO_2_. The final concentration of *Rikenellaceae* was 2 × 10^8^ viable c.f.u. per 100 μL and hypoxanthine (200 mg/kg, Sigma, Germany) was dissolved in double distilled water^29^. Mice first received antibiotic treatment (same as FMT) and were then treated by oral gavage with 100 μL of either *Rikenellaceae* or hypoxanthine suspension three times a week for 4 weeks. Reinforced Clostridial Medium or double distilled water was used as a vehicle control, respectively. 2 days after the last administration, recipients were euthanized to analyze the changes in the hematopoietic system. To examine the impact of hypoxanthine on HSCs, we exposed bone marrow cells to direct co-culture with hypoxanthine at a concentration of 100 pg/mL for a period of 3 days.

### Cell culture

Mouse bone marrow cells were harvested by flushing the mice’s tibia and femur in phosphate buffered saline (PBS) with 2% fetal bovine serum (GIBCO). Harvested cells were grown into 96-well u-bottom plates containing freshly made HSC culture medium (StemSpanTM SFEM, Stemcell Tec.) with SCF (50 ng/mL; PeproTech) and TPO (50 ng/mL; PeproTech), at 37 °C with 5% CO_2_. For HSC culture, the medium was changed every 3 days by manually removing half of the conditioned medium and replacing it with fresh medium^60^. To assess the effects of WNT10A, IL-17, TNF and NF-kappa B on hematopoiesis, we cultured HSCs in a basic medium and supplemented them with related proteins (10 ng/mL; Cosmo Bio, USA) or PBS as a control for two days, followed by flow cytometry analysis. Different concentrations of PS were added to the medium and tested using CCK-8 and FACS to detect the effect of MPs on cultured HSCs.

### RNA-seq

1 × 10^4^ HSCs were obtained in triplicate from mouse bone marrow cells from the ctrl or PS_H_ group by flow cytometry sorting and RNA was extracted with RNAiso Plus (Takara, Japan) according to the manufacturer’s protocol. The concentration and integrity of RNA were examined by Qubit 2.0 and Agilent 2100 (Novogene, China), respectively. Oligo (dT)-coated magnetic beads (Novogene, China) were used to enrich eukaryotic mRNA. After cDNA synthesis and PCR amplification, the PCR product was purified using AMPure XP beads (Novogene, China) to obtain the final library. The Illumina high-throughput sequencing platform NovaSeq 6000 was used for sequencing. Analysis of gene expression was calculated by R or the DESeq2 package^61^. Detailed information regarding RNA-seq is listed in Supplementary Table S3.

### Quantification of gene expression by qPCR

For RNA expression analysis, total RNA from bone marrow cells was extracted using Trizol (Invitrogen, US) and resuspended in nuclease-free water. Reverse transcription was performed using the QuantiTect Reverse Transcription kit (Qiagen NV). qPCR was conducted using cDNA, primers and SYBR-green (Takara, Japan) in 20 μL using the ABI 7500 Q-PCR system^62^. Results were calculated using the RQ value (RQ = 2^−ΔΔCt^). Mouse Actin was chosen as the normalization control. Gene-specific primer sequences are shown in Supplementary Table S7.

### ELISA

Bone marrow and *Rikenellaceae* supernatant in different groups were obtained by centrifugation. Fecal supernatant was obtained from human samples. Hypoxanthine (LANSO, China) and WNT10A (EIAab, China) were measured by ELISA with respective kits according to the manufacturer’s protocols.

### Clinical patient samples

Human feces and peripheral blood samples were obtained from 14 subjects who provided grafts for HSCT patients. They were divided into graft success group and graft failure (GS)/poor graft function (GF/PGF) group, with 7 participants in each group. Research involving humans was approved by the Clinical Research Ethics Committee of the First Affiliated Hospital, College of Medicine, Zhejiang University (IIT20230067B). All participants read and signed the informed consent. Detailed information on patients was listed in Supplementary Table S4.

### LDIR

The Agilent 8700 Laser Direct Infrared Imaging system was utilized for fast and automated analysis of MPs in feces received from donors. An excessive nitric acid concentration (68%) was added to the sample and heated to dissolve the protein. Large particles were first intercepted with a large aperture filter and then filtered by vacuum extraction. After rinsing with ultra-pure water and ethanol several times, the materials, including MPs, were dispersed in the ethanol solution. The LDIR test was carried out when the ethanol was completely volatilized^63^. The sample of MPs was positioned on the standard sample stage. The stage was then put into the sample stage, and the Agilent Clarity was initiated to advance the sample stage into the sample chamber. The software rapidly scanned the chosen test area using a constant wave number of 1800 cm^−1^, and accurately detected and pinpointed the particles within the selected area. The unoccupied area devoid of particles was automatically designated as the background. The background spectrum was gathered and readjusted, followed by the visualization of detected particles and the collection of the whole infrared spectrum. After obtaining the particle spectrum, the spectrum library was utilized to carry out qualitative analysis automatically, including the inclusion picture, size, and area of each particle. The test was supported by Shanghai WEIPU Testing Technology Group.

### Py-GC/MS

MPs in peripheral blood from donors were tested by Py-GC/MS. Nitric acid was added to samples for digestion at 110 °C for 1−2 h, and then used deionized water to make the solution weakly acidic. After concentration, the solution was dribbled into the sampling crucible of Py-GCMS and tested when the solvent in the crucible was completely volatilized^17^. Various standards of MPs were prepared and analyzed using Py-GCMS in order to construct the quantitative curve. PY-3030D Frontier was employed for lysis, with a lysis temperature set at 550 °C. The chromatographic column dimensions were 30 m in length, 0.25 mm inner diameter, and 0.25 μm film thickness. The sample was subjected to a heat preservation period of 2 min at 40 °C, followed by a gradual increase in temperature at a rate of around 20 °C per minute until it reached 320 °C. The sample was maintained at this temperature for 14 min and the entire process takes a total of 30 min. The carrier gas utilized was helium, with the ion source temperature of 230 °C. The split ratio employed was 5:1, and the m/z scan range spanned from 40 to 600^64^. The experiment was supported by Shanghai WEIPU Testing Technology Group.

### Statistical analysis

Each animal experiment was tested using at least 5−6 replicates and each in vitro experiment was at least three replicates. Specific replication details are provided in relevant figure captions. Statistical significance was ascertained through unpaired two-tailed *t*-tests by GraphPad Prism when the *P* value was less than 0.05. Error bars in all figures indicate the standard deviation (SD).

### Supplementary information

Supplementary Table S1. Bacterial OTUs taxa list compared in ctrl, PSL and PSH group.

Supplementary Table S2: Differential expression of metabolites taxa compared between ctrl and PSH group.

Supplementary Table S3: High-throughput RNA-seq revealed the FPKMs of all transcripts in LT-HSCs sorted from ctrl and MPs-treated mice.

Supplementary Table S4. Clinical chateristics and information of HSCT donors and recipients in our cohort.

Supplementary Table S5. Bacterial OTUs taxa list compared in GS and GF/PGF group.

Supplementary Table S6. List of antibodies used in this study.

Supplementary Table S7. Primer sets for qPCR analyses used in this study.

Supplementary Fig. S1 Physical characterization of microplastics.

Supplementary Fig. S2 Distribution of microplastics in vivo

Supplementary Fig. S3 Short-term ingestion of microplastics has almost no effect on the hematopoietic system.

Supplementary Fig. S4 Representative flow cytometry images of long-term ingestion model and analysis of graft reconstruction of BM cells.

Supplementary Fig. S5 Long-term ingestion of PMMA or PE also damages hematopoietic system.

Supplementary Fig. S6 Microplastics exhibit no toxicity on LT-HSCs during in vitro culture.

Supplementary Fig. S7 Hematopoietic stem and progenitor cells are damaged in Recip-PS group after FMT.

Supplementary Fig. S8 Rikenellaceae administration restored HSPC functions in the mice with microplastics.

Supplementary Fig. S9 Hypoxanthine treatment partially mitigates the damage of HSPCs in the PSH mice.

Supplementary Fig. S10 Wnt signaling pathway is beneficial to the recovery of HSCs function and microplastics play a detrimental role in HSC transplantation.

## Data Availability

All the sequencing data have been submitted to the National Genomics Data Center, https://ngdc.cncb.ac.cn/. The datasets of mice generated during this study have been deposited in the Genome Sequence Archive (GSA: CRA010086, Shared URL: https://ngdc.cncb.ac.cn/gsa/s/n308TgY6; GSA: CRA010069, Shared URL: https://ngdc.cncb.ac.cn/gsa/s/Z3XJaqeA) and Open Archive for Miscellaneous Data (OMIX003159, Shared URL: https://ngdc.cncb.ac.cn/omix/preview/nrD7b5Mw). The sequencing data of gut microbiota from humans are accessible at GSA for human database with accession number HRA004119 (Shared URL: https://ngdc.cncb.ac.cn/gsa-human/s/5SMsyfid).
