# Supplementary material for: Microplastics dampen the self-renewal of hematopoietic stem cells by disrupting the gut microbiota-hypoxanthine-Wnt axis
Source: Cell Discov. 2024 Mar 29;10:35. doi: 10.1038/s41421-024-00665-0 (PMC10978833; doi:10.1038/s41421-024-00665-0)
Supplement: Supplementary file 8 — Supplementary Fig. S1 Physical characterization of microplastics. [file 41421_2024_665_MOESM8_ESM.pdf]

Supplementary Fig. S1

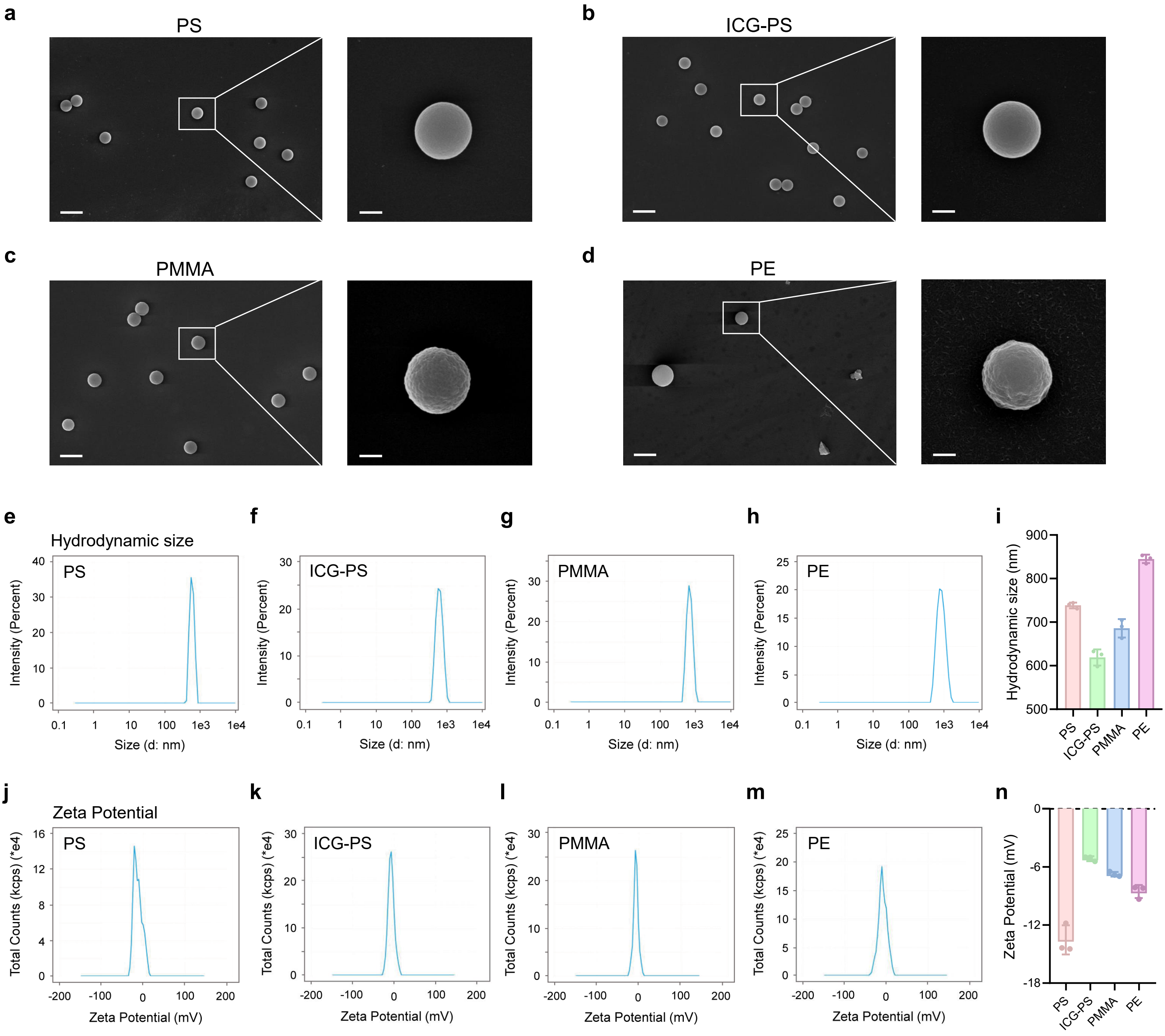

Supplementary Fig. S1 | Physical characterization of microplastics.

a-d, Representative scanning electron microscope (SEM) image of PS (**a**), ICG-PS (**b**), PMMA (**c**) and PE (**d**). Scale bar, 1µm (left) or 200nm (right). **e-h**, Representative Dynamic light scattering (DLS) characterization for PS (**e**), ICG-PS (**f**), PMMA (**g**) and PE (**h**). i, Statistical plot of hydrodynamic size. **j-m**, Representative zeta potential characterization for PS (**j**), ICG-PS (**k**), PMMA (**l**) and PE (**m**). **n**, Statistical plot of zeta potential. At least three independent experiments were taken (n=3).
