# Supplementary material for: Microplastics dampen the self-renewal of hematopoietic stem cells by disrupting the gut microbiota-hypoxanthine-Wnt axis
Source: Cell Discov. 2024 Mar 29;10:35. doi: 10.1038/s41421-024-00665-0 (PMC10978833; doi:10.1038/s41421-024-00665-0)
Supplement: Supplementary file 9 — Supplementary Fig. S2 Distribution of microplastics in vivo [file 41421_2024_665_MOESM9_ESM.pdf]

Supplementary Fig. S2

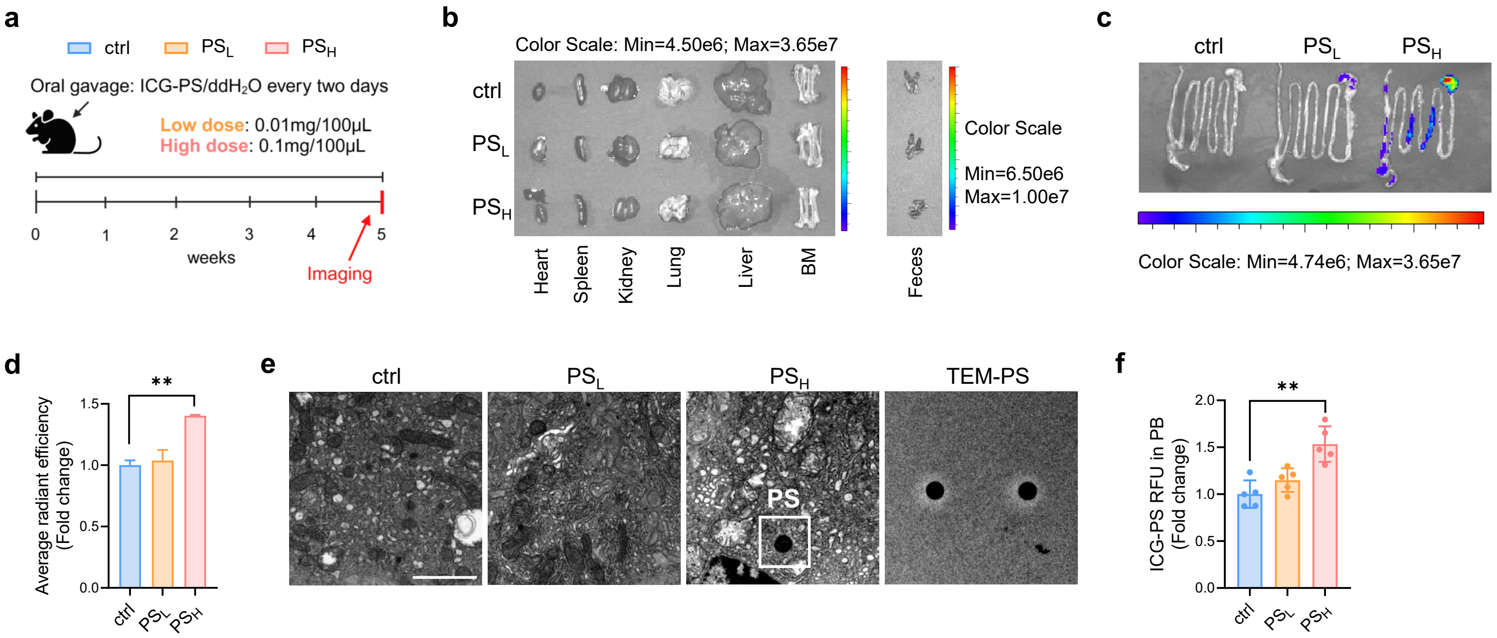

Supplementary Fig. S2 | Distribution of microplastics in *vivo*.

a, Schematic outlining mouse model for ICG-PS treatment. **b**, Distribution of ICG-PS in main tissues and feces after 5weeks gavage. Color scale: Min=4.50e6; Max=3.65e7 (left); Min=6e6; Max=1e7 (right). **c**, Distribution of ICG-PS in gastrointestinal tissues after 5 weeks gavage. Color scale: Min=4.74e6; Max=3.65e7. **d**, Average radiant efficiency of ICG-PS. **e**, Representative transmission electron microscope (TEM) images of intestinal cell and ICG-PS. Scale bar, 2μm (n ≥ 3 per group). **f**, Body weight of each mouse of the three groups. The data are shown as the mean ± SD. Unpaired two-tailed t-test, *$P < 0.05$, **$P < 0.01$.
