# Supplementary material for: Microplastics dampen the self-renewal of hematopoietic stem cells by disrupting the gut microbiota-hypoxanthine-Wnt axis
Source: Cell Discov. 2024 Mar 29;10:35. doi: 10.1038/s41421-024-00665-0 (PMC10978833; doi:10.1038/s41421-024-00665-0)
Supplement: Supplementary file 10 — Supplementary Fig. S3 Short-term ingestion of microplastics has almost no effect on the hematopoietic system. [file 41421_2024_665_MOESM10_ESM.pdf]

Supplementary Fig. S3

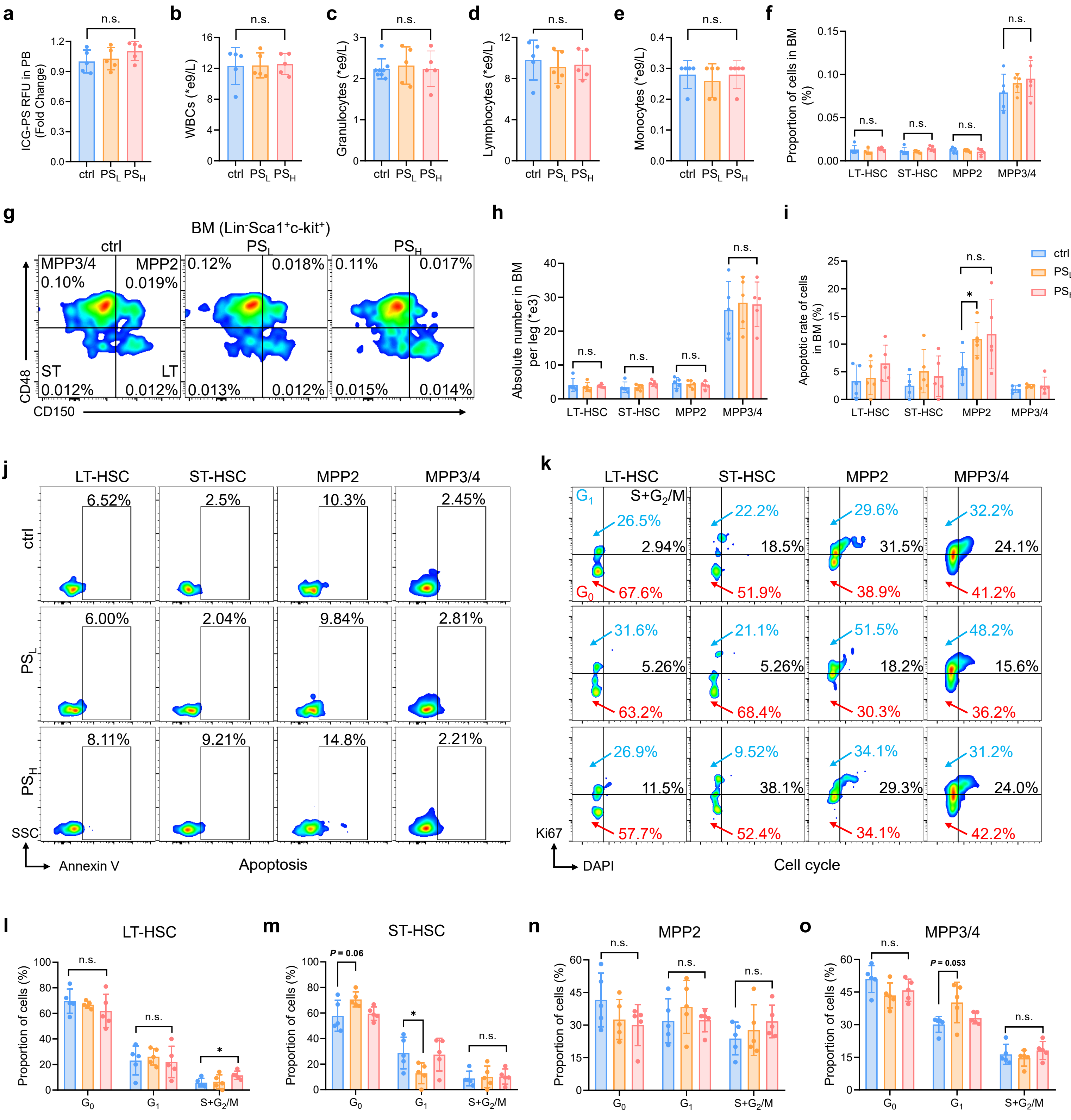

Supplementary Fig. S3 | Short-term ingestion of microplastics has almost no effect on the hematopoietic system.

a, Relative fluorescence intensity of ICG-PS in peripheral blood (n = 5 per group). **b-e**, Hemogram of ctrl, PS_L and PS_H mice including WBCs (**b**), granulocytes (**c**), lymphocytes (**d**) and monocytes (**e**). **f-g**, Proportion (**f**) and representative FACS images (**g**) of HSPCs in BM. **h**, Absolute cell number. **i**, Apoptotic rate of HSPCs. **j-k**, Representative FACS images of apoptosis (**j**) and cell cycle (**k**) of LT-HSCs. **l-o**, Percentage of cells in individual cycle phases of LT-HSCs (**l**), ST-HSCs (**m**), MPP2s (**n**) and MPP3/4s (**o**). Error bars indicate SD, unpaired two-tailed t-test, n.s., not significant.
