# Supplementary material for: Microplastics dampen the self-renewal of hematopoietic stem cells by disrupting the gut microbiota-hypoxanthine-Wnt axis
Source: Cell Discov. 2024 Mar 29;10:35. doi: 10.1038/s41421-024-00665-0 (PMC10978833; doi:10.1038/s41421-024-00665-0)
Supplement: Supplementary file 11 — Supplementary Fig. S4 Representative flow cytometry images of long-term ingestion model and analysis of graft reconstruction of BM cells. [file 41421_2024_665_MOESM11_ESM.pdf]

Supplementary Fig. S4

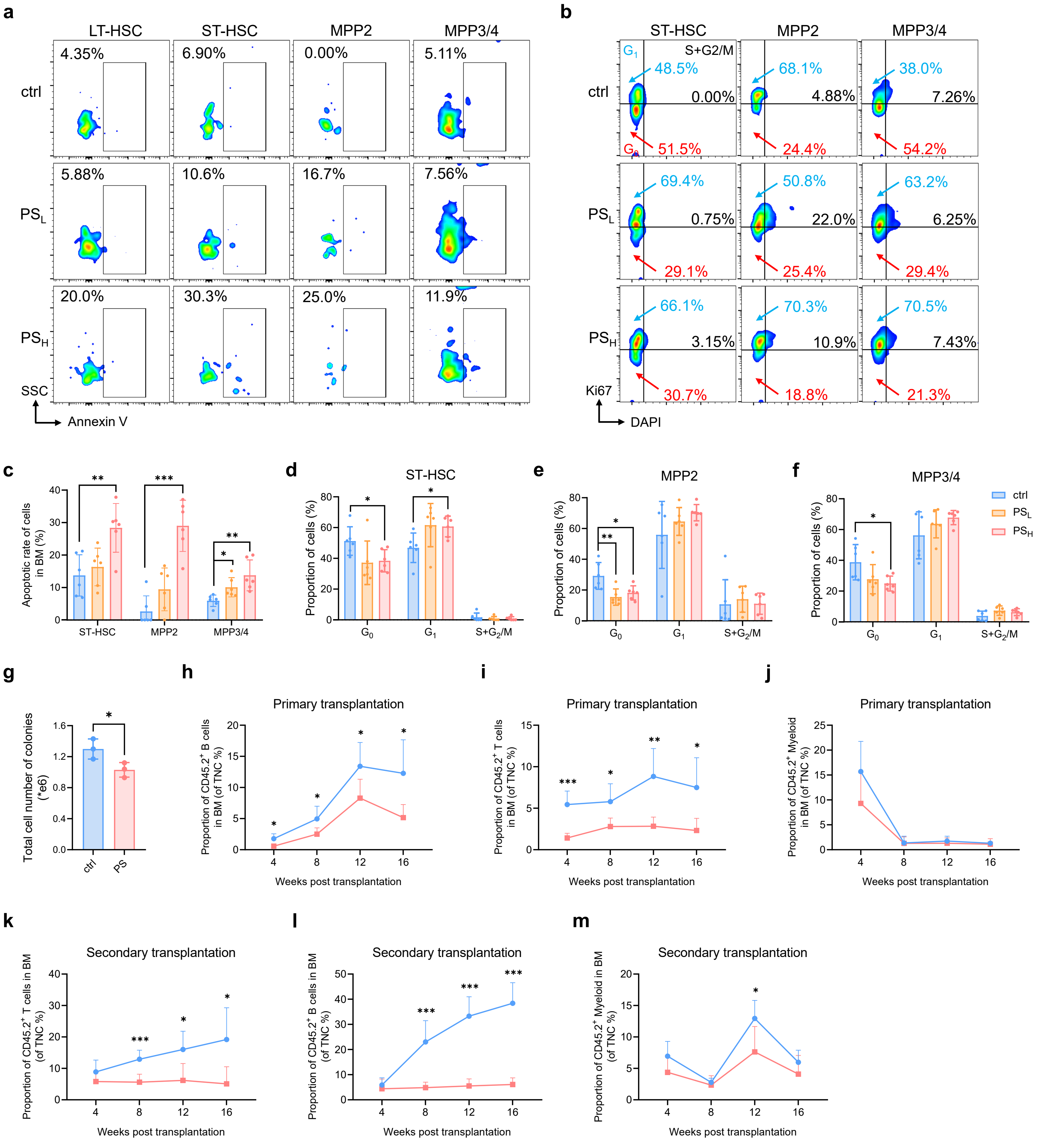

Supplementary Fig. S4 | Representative flow cytometry images of long-term ingestion model and analysis of graft reconstruction of BM cells.

a-b, Representative FACS images of apoptosis (**a**) and cell cycle (**b**). **c**, Apoptotic rate of ST-HSCs, MPP2s and MPP3/4s. **d-f**, Percentage of cells in individual cycle phases of ST-HSCs (**d**), MPP2s (**e**) and MPP3/4s (**f**). **g**, Total cell number of colonies. **h-j**, Proportion of CD45.2$^+$ T cells (**h**), B cells (**i**) and myeloid cells (**j**) in primary transplantation. **k-m**, Proportion of CD45.2$^+$ T cells (**k**), B cells (**l**) and myeloid cells (**m**) in secondary transplantation. Error bars indicate SD, unpaired two-tailed t-test. **P* < 0.05, ***P* < 0.01, ****P* < 0.001.
