# Supplementary material for: Microplastics dampen the self-renewal of hematopoietic stem cells by disrupting the gut microbiota-hypoxanthine-Wnt axis
Source: Cell Discov. 2024 Mar 29;10:35. doi: 10.1038/s41421-024-00665-0 (PMC10978833; doi:10.1038/s41421-024-00665-0)
Supplement: Supplementary file 12 — Supplementary Fig. S5 Long-term ingestion of PMMA or PE also damages hematopoietic system. [file 41421_2024_665_MOESM12_ESM.pdf]

Supplementary Fig. S5

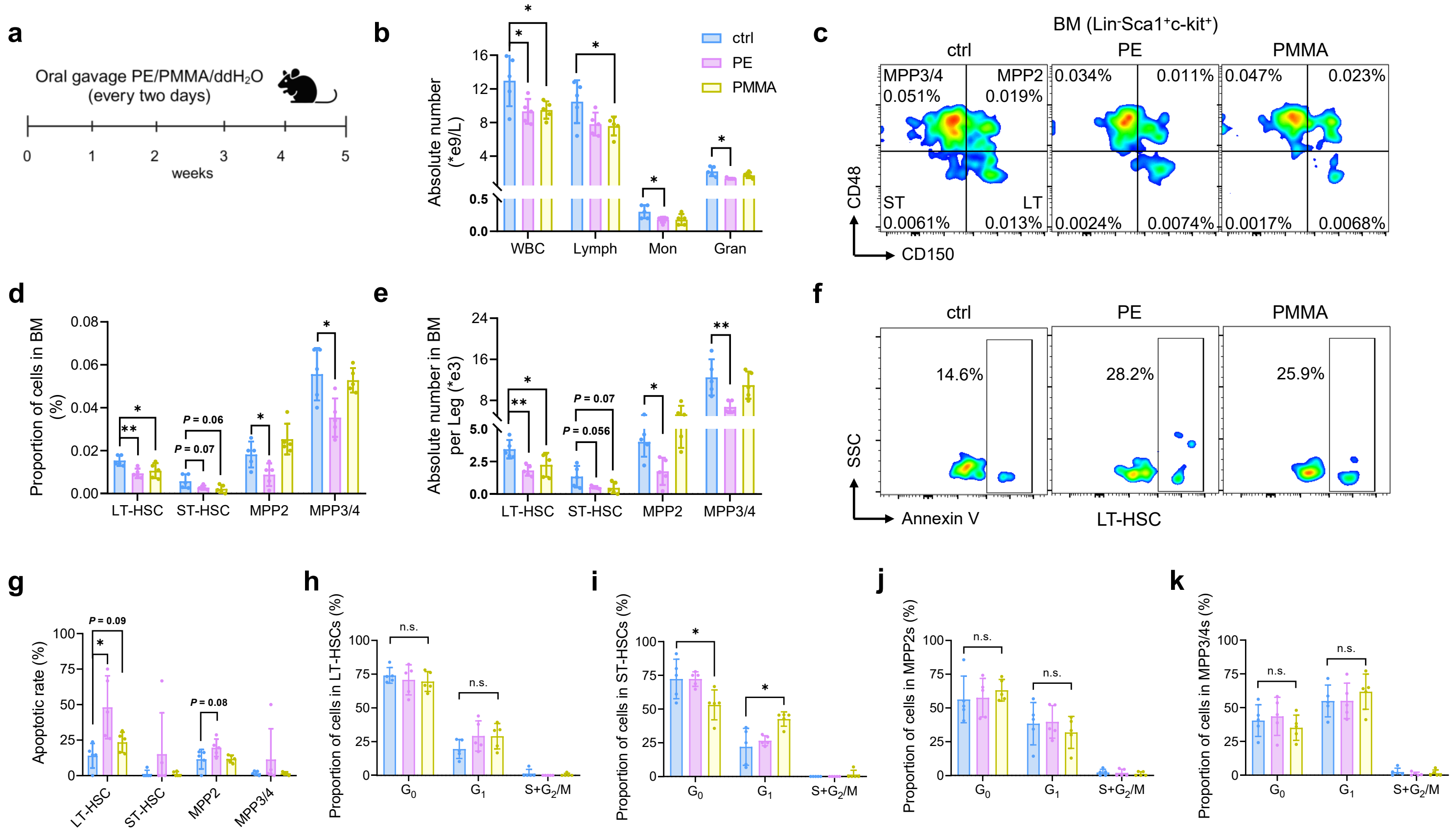

Supplementary Fig. S5 | Long-term ingestion of PMMA or PE also damages hematopoietic system.

a, Schematic of the experimental design. **b**, Hemogram of ctrl, PMMA and PE mice (n = 5 per group). **c-e**, Representative FACS images (**c**), proportion (**d**) and absolute number of HSPCs (**e**) in BM. f-g, Representative FACS images (**f**) and proportion of apoptosis (**g**) in LT-HSCs. **h-k**, Proportion of cells in individual cycle phases of LT-HSCs (**h**), ST-HSCs (**i**), MPP2s (**j**) and MPP3/4s (**k**). Error bars indicate SD, unpaired two-tailed t-test. **P* < 0.05, ***P* < 0.01, n.s., not significant.
