# Supplementary material for: Microplastics dampen the self-renewal of hematopoietic stem cells by disrupting the gut microbiota-hypoxanthine-Wnt axis
Source: Cell Discov. 2024 Mar 29;10:35. doi: 10.1038/s41421-024-00665-0 (PMC10978833; doi:10.1038/s41421-024-00665-0)
Supplement: Supplementary file 13 — Supplementary Fig. S6 Microplastics exhibit no toxicity on LT-HSCs during in vitro culture. [file 41421_2024_665_MOESM13_ESM.pdf]

Supplementary Fig. S6

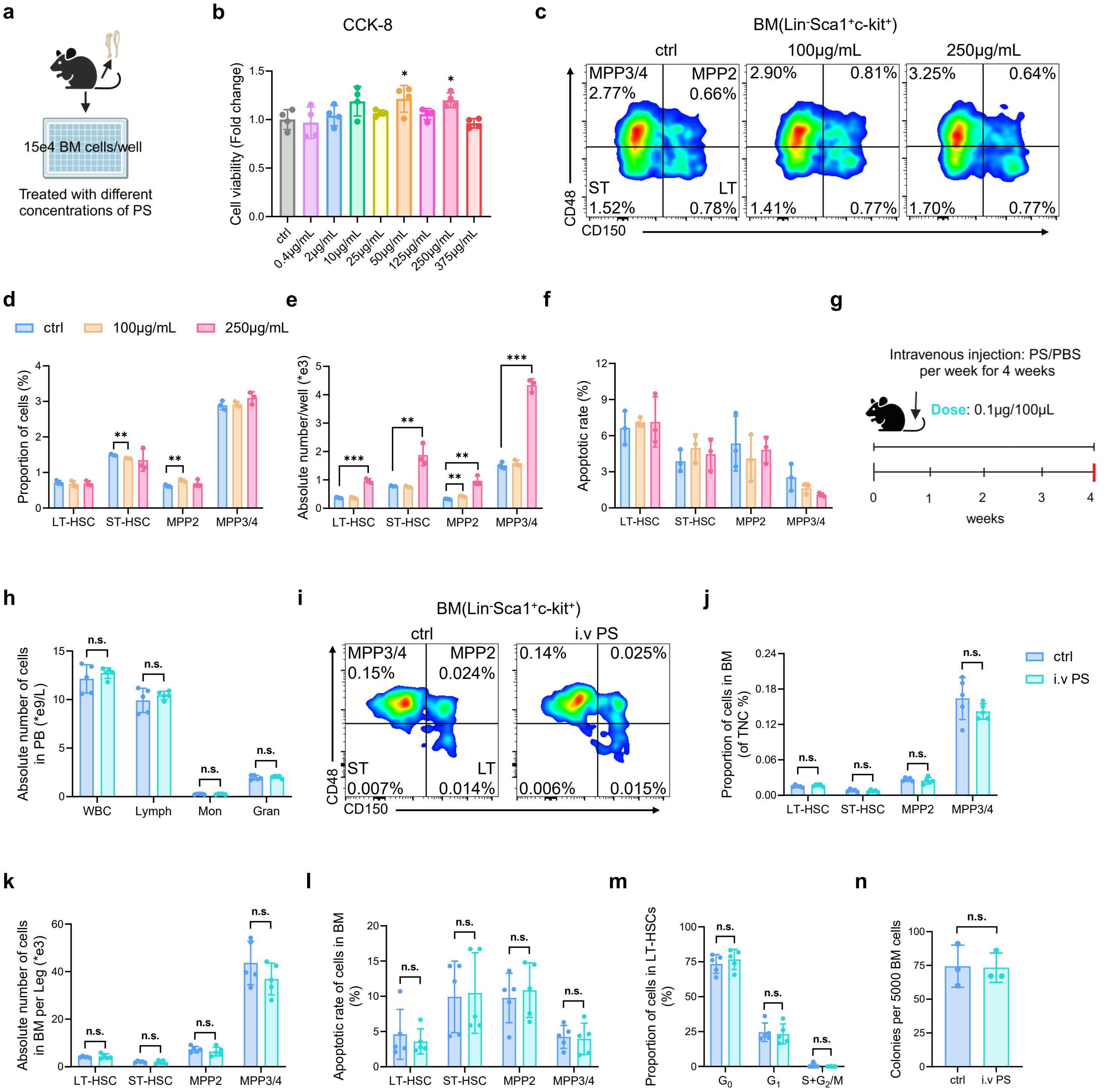

Supplementary Fig. S6 | Microplastics exhibit no toxicity on LT-HSCs during in vitro culture.

a, Schematic of the in vitro BM cell culture. **b**, Cell viability of BM cells through 7d culture (n = 3 per group). **c-e**, Representative FACS images (**c**), proportion (**d**) and absolute number of HSPCs (**e**) in 14d culture in vitro. **f**, Apoptotic rate of LT-HSCs in vitro culture for 14 days. **g**, Schematic of intravenous injection MPs to mice (n = 5 per group). **h**, Hemogram of mice including WBCs, granulocytes, lymphocytes and monocytes. **i-k**, Representative FACS images (**i**), proportion (**j**) and absolute number of HSPCs (**k**) in mice. **l**, Apoptosis of HSPCs in bone marrow. **m**, Cell cycle of LT-HSCs in the two groups. **n**, Number of colonies formed by 5000 bone marrow cells. Error bars indicate SD, unpaired two-tailed t-test. *$P < 0.05$, **$P < 0.01$, ***$P < 0.001$.
