# Supplementary material for: Microplastics dampen the self-renewal of hematopoietic stem cells by disrupting the gut microbiota-hypoxanthine-Wnt axis
Source: Cell Discov. 2024 Mar 29;10:35. doi: 10.1038/s41421-024-00665-0 (PMC10978833; doi:10.1038/s41421-024-00665-0)
Supplement: Supplementary file 14 — Supplementary Fig. S7 Hematopoietic stem and progenitor cells are damaged in Recip-PS group after FMT. [file 41421_2024_665_MOESM14_ESM.pdf]

Supplementary Fig. S7

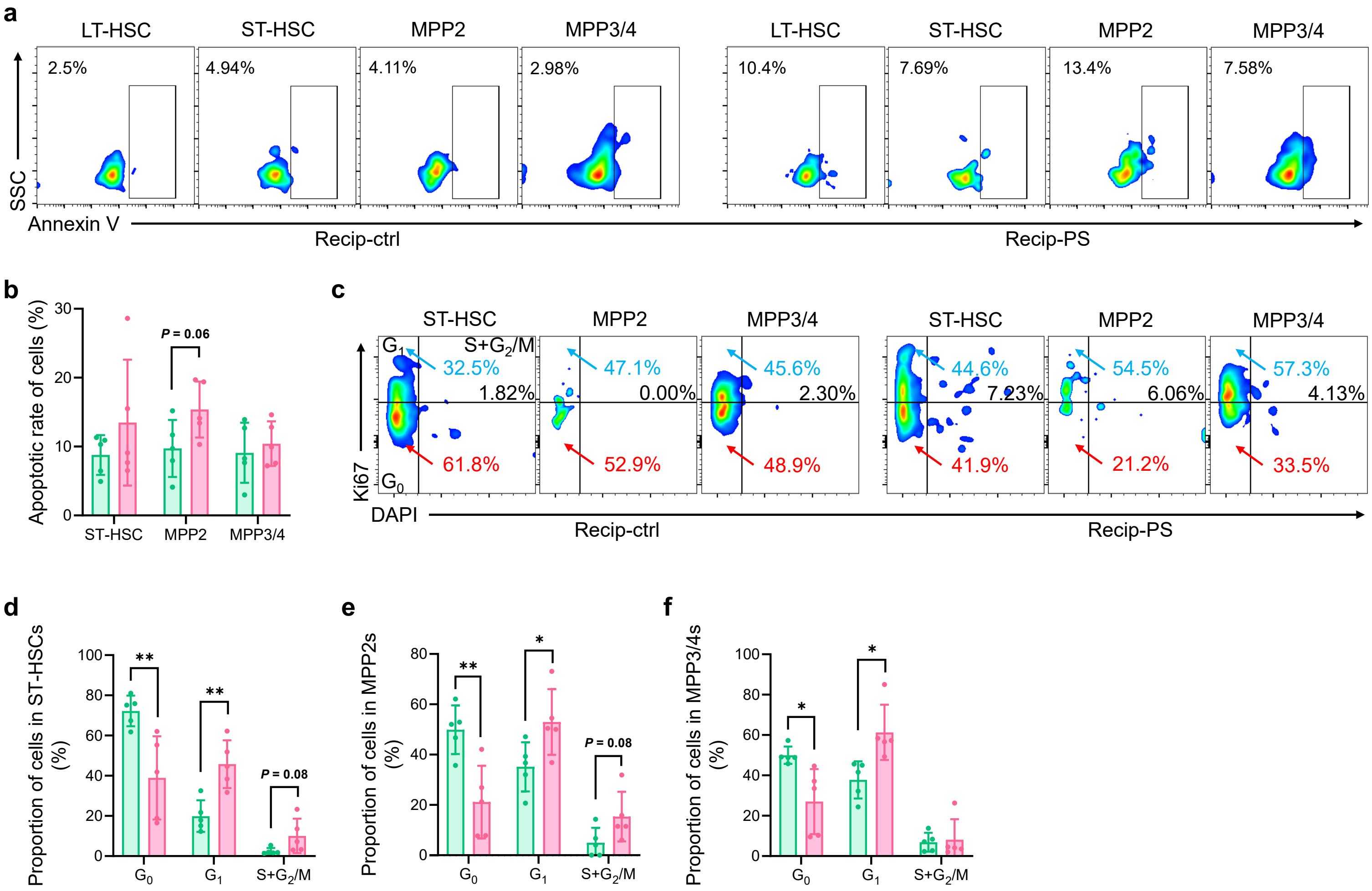

Supplementary Fig. S7 | Hematopoietic stem and progenitor cells are damaged in Recip-PS group after FMT.

a-b, Representative flow cytometry plots (**a**) and proportion of apoptotic rate in HSPCs (**b**) (n = 5 per group). **c**, Representative flow cytometry plots of cell cycle in HSPCs. **d-f**, Proportion of cells in individual cycle phases of ST-HSCs (**d**), MPP2s (**e**) and MPP3/4s (**f**). Error bars indicate SD, unpaired two-tailed t-test. *$P < 0.05$, **$P < 0.01$, ***$P < 0.001$.
