# Supplementary material for: Microplastics dampen the self-renewal of hematopoietic stem cells by disrupting the gut microbiota-hypoxanthine-Wnt axis
Source: Cell Discov. 2024 Mar 29;10:35. doi: 10.1038/s41421-024-00665-0 (PMC10978833; doi:10.1038/s41421-024-00665-0)
Supplement: Supplementary file 15 — Supplementary Fig. S8 Rikenellaceae administration restored HSPC functions in the mice with microplastics. [file 41421_2024_665_MOESM15_ESM.pdf]

Supplementary Fig. S8

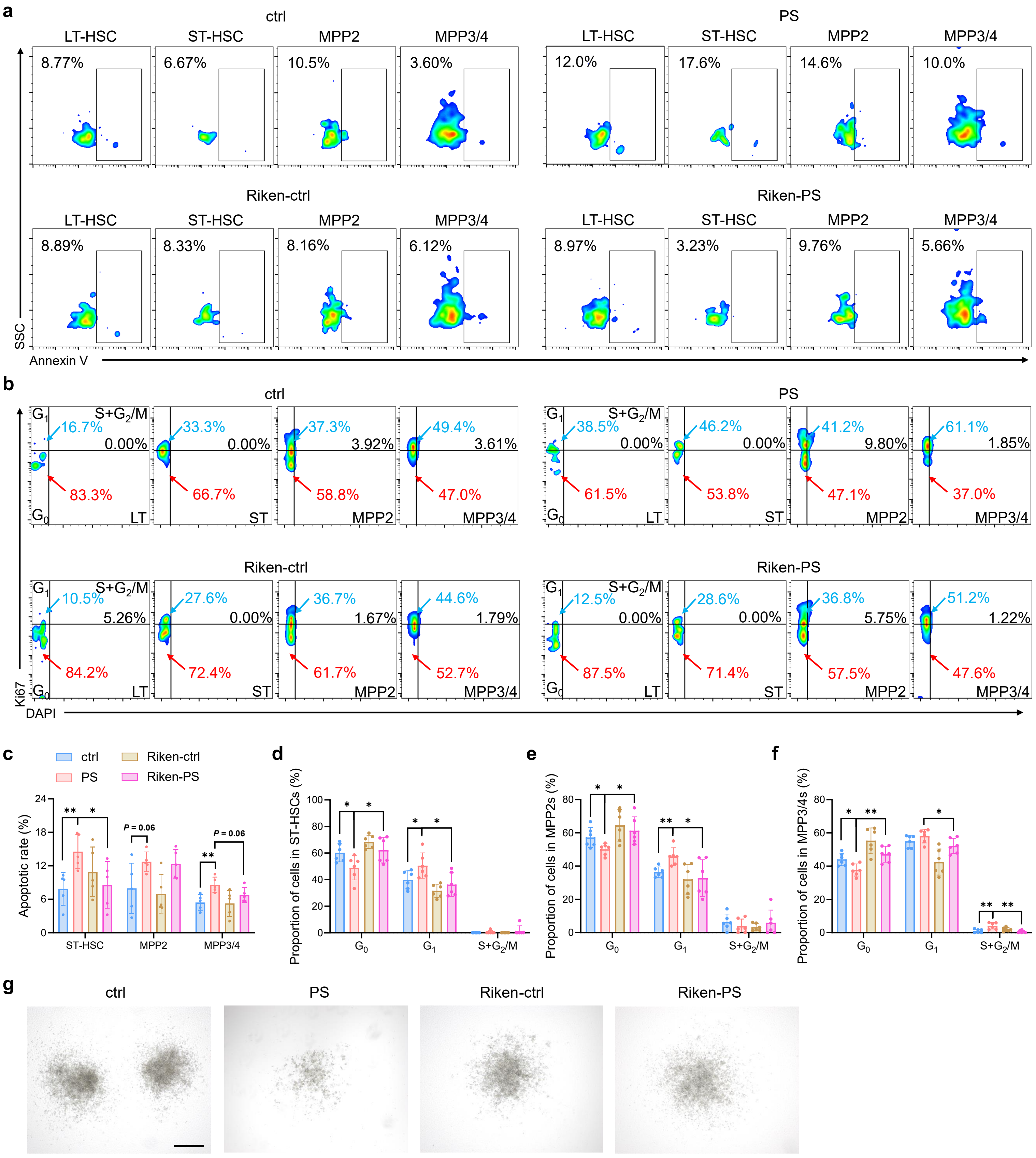

Supplementary Fig. S8 | *Rikenellaceae* administration restored HSPC functions in the mice with microplastics.

a-b, Representative flow cytometry analysis of apoptosis (**a**) and cell cycle profiles (**b**) (n = 5 per group). **c**, Apoptotic rate of HSPCs. **d-f**, FACS quantification of cell cycle in ST-HSCs (**d**), MPP2s (**e**) and MPP3/4s (**f**). **g**, Representative picture of colonies in ctrl, PS, Riken-ctrl and Riken-PS group. Error bars indicate SD, unpaired two-tailed t-test. $*P < 0.05$, $**P < 0.01$, $***P < 0.001$.
