# Supplementary material for: Microplastics dampen the self-renewal of hematopoietic stem cells by disrupting the gut microbiota-hypoxanthine-Wnt axis
Source: Cell Discov. 2024 Mar 29;10:35. doi: 10.1038/s41421-024-00665-0 (PMC10978833; doi:10.1038/s41421-024-00665-0)
Supplement: Supplementary file 16 — Supplementary Fig. S9 Hypoxanthine treatment partially mitigates the damage of HSPCs in the PSH mice. [file 41421_2024_665_MOESM16_ESM.pdf]

Supplementary Fig. S9

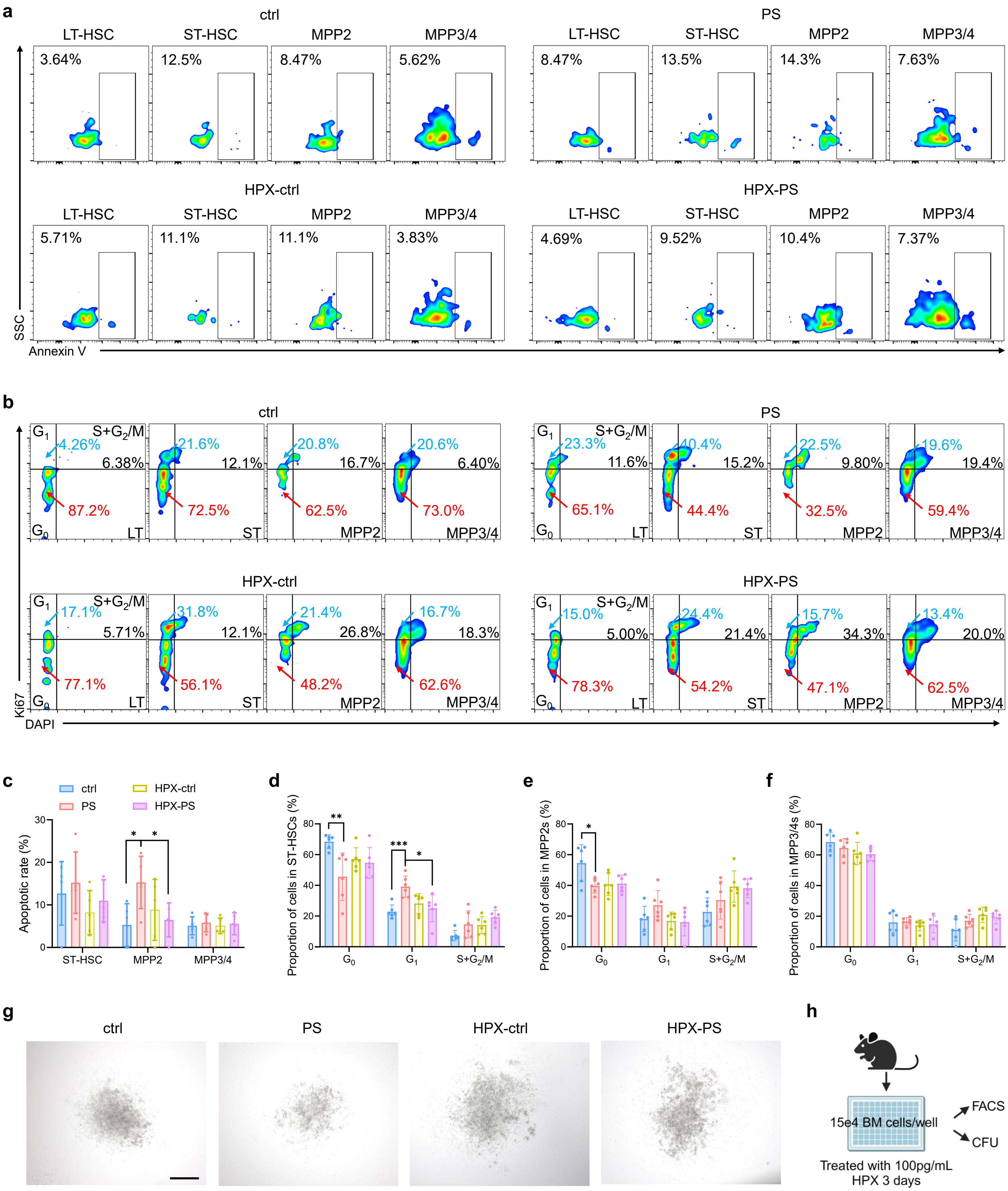

Supplementary Fig. S9

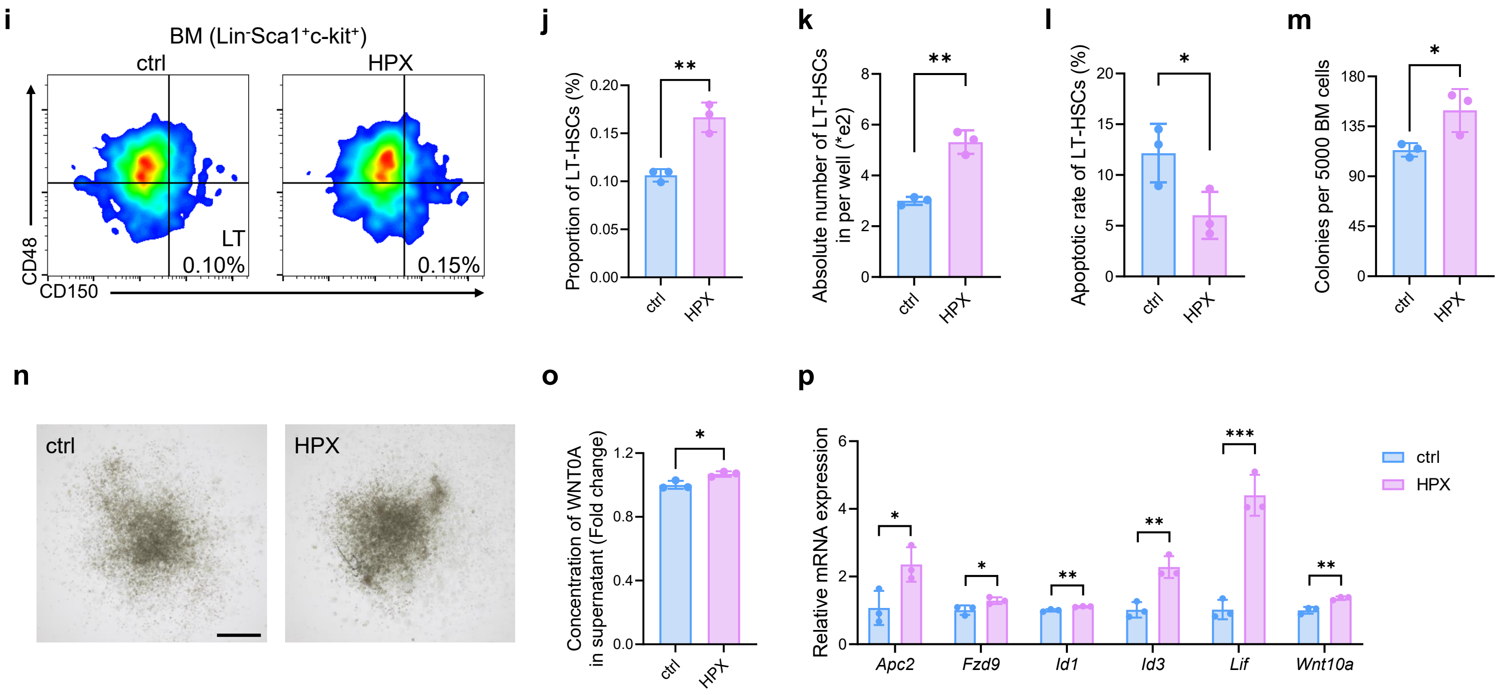

Supplementary Fig. S9 | Hypoxanthine treatment partially mitigates the damage of HSPCs in the PS_H mice.

a-b, Representative FACS images of apoptosis (a) and cell cycle profiles (**b**) (n = 5 per group). **c**, Proportion of apoptosis in HSPCs. **d-f**, FACS analysis of cell cycle in ST-HSCs (**d**), MPP2s (**e**) and MPP3/4s (**f**). **g**, Representative images of colonies in ctrl, PS, HPX-ctrl and HPX-PS group. **h**, Schematic of the hypoxanthine culture in vitro to BM cells. **i-k**, Representative FACS images (**i**), proportion (**j**) and absolute number of cells (**k**) in mice. **l**, Apoptosis of LT-HSCs. **m-n**, Number of colonies formed by 5000 bone marrow cells (**m**) and representative picture of colonies (**n**). **o**, Concentration of WNT10A in the supernatant. **p**, Relative expression of genes in the Wnt signaling pathway. Error bars indicate SD, unpaired two-tailed t-test. $*P < 0.05$, $**P < 0.01$, $***P < 0.001$.
