# Supplementary material for: Microplastics dampen the self-renewal of hematopoietic stem cells by disrupting the gut microbiota-hypoxanthine-Wnt axis
Source: Cell Discov. 2024 Mar 29;10:35. doi: 10.1038/s41421-024-00665-0 (PMC10978833; doi:10.1038/s41421-024-00665-0)
Supplement: Supplementary file 17 — Supplementary Fig. S10 Wnt signaling pathway is beneficial to the recovery of HSCs function and microplastics play a detrimental role in HSC transplantation. [file 41421_2024_665_MOESM17_ESM.pdf]

Supplementary Fig. S10

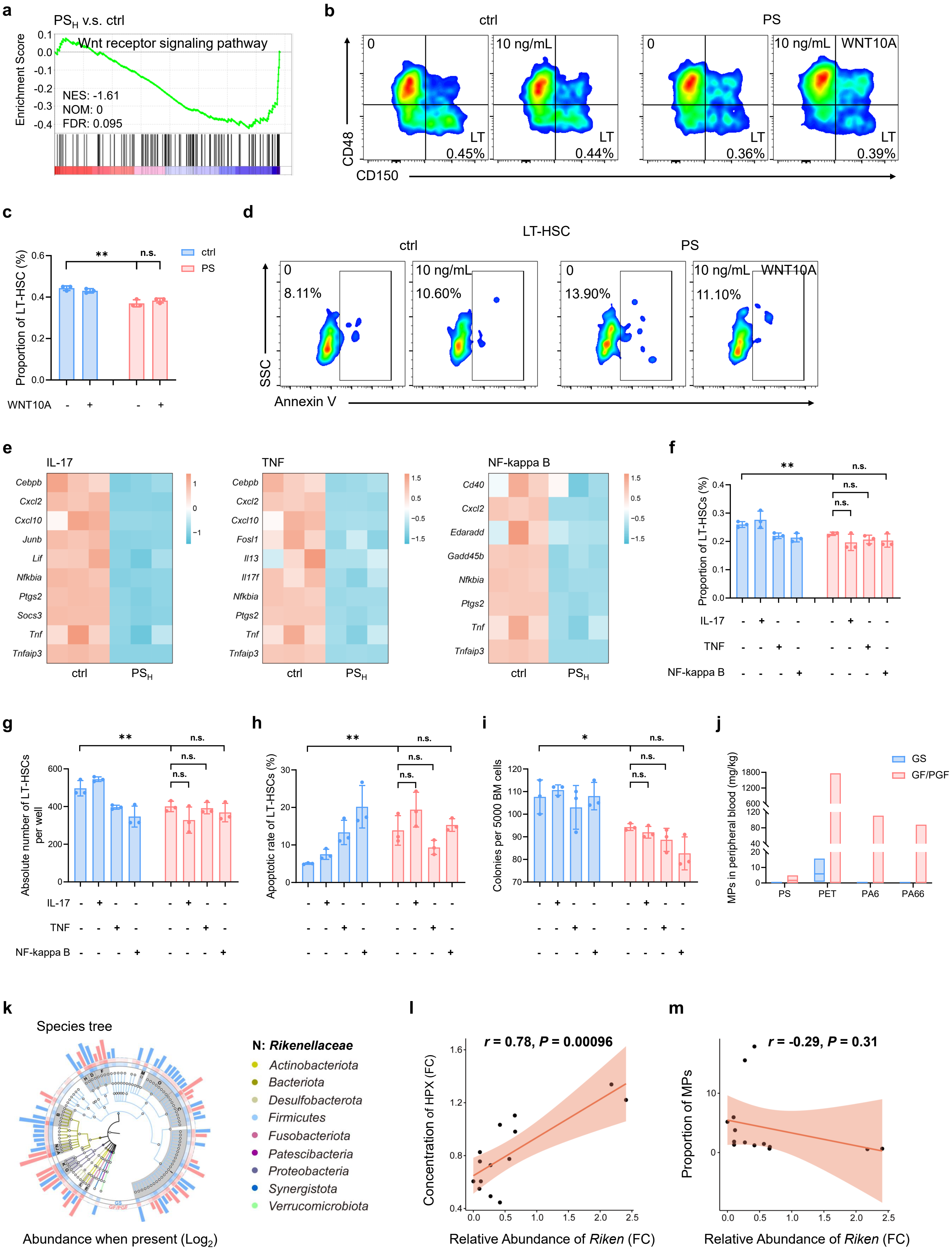

Supplementary Fig. S10

n

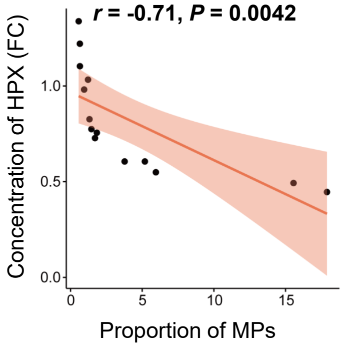

o

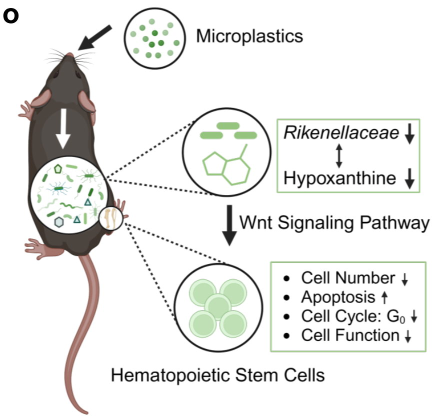

Supplementary Fig. S10 | Wnt signaling pathway is beneficial to the recovery of HSCs function and microplastics play a detrimental role in HSC transplantation.

a, Gene set enrichment analysis (GSEA) in LT-HSCs from the PS_H group v.s. the ctrl group for “Wnt receptor signaling pathway”. **b-c**, Representative flow cytometry images (**b**) and proportion of cells (**c**) during cell culture in vitro. **d**, Representative flow cytometry images of apoptosis. **e**, Relative expression of genes in the IL-17, TNF and NF-kappa B signaling pathways. **f-g**, Proportion (**f**) and absolute number (**g**) of LT-HSCs after being treated with IL-17, TNF or NF-kappa B. **h**, Apoptotic rate of LT-HSCs. **i**, Number of colonies formed by 5000 bone marrow cells. (n = 3 per group) **j**, Detection of microplastics in peripheral blood. **k**, Species tree in feces of donor provided BM cells. **l-n**, Correlation between relative abundance of *Rikenellaceae* and concentration of hypoxanthine in feces (**l**), relative abundance of *Rikenellaceae* and proportion of microplastics in feces (**m**) or concentration of hypoxanthine and proportion of microplastics in feces (**n**). The Pearson correlation coefficient (r) and empirical P value were also shown. **o**, Proposed schema of the microplastics mechanism to hematopoietic stem cells in mice (n = 7 per group). Error bars indicate SD, unpaired two-tailed t-test. $^{**}P < 0.01$.
